# *Gentiana capitata* Buch.–Ham. ex D.Don Cell Suspension Culture as a New Source of Isosaponarin and 3,7,8-Trimethoxy-9-oxo-9H-xanthen-1-yl 6-*O*-*β*-D-ribopyranosyl-*β*-D-allopyranoside and Their Effect on PC-12 Cell Viability

**DOI:** 10.3390/ijms25168576

**Published:** 2024-08-06

**Authors:** Zuzanna Czarnomska, Michał Markowski, Ewa K. Nawrocka, Wiktor Koźmiński, Agnieszka Bazylko, Wojciech J. Szypuła

**Affiliations:** 1 Department of Pharmaceutical Biology, Faculty of Pharmacy, Medical University of Warsaw, ul. Banacha 1, 02-097 Warsaw, Poland; zuzannaczarnomska@gmail.com (Z.C.); markowski.mic@gmail.com (M.M.); agnieszka.bazylko@wum.edu.pl (A.B.); 2 Centre of New Technologies, University of Warsaw, ul. Banacha 2C, 02-097 Warsaw, Poland; ewanawrocka1993@gmail.com; 3 Biological and Chemical Research Centre, Faculty of Chemistry, University of Warsaw, ul. Żwirki i Wigury 101, 02-089 Warsaw, Poland; kozmin@chem.uw.edu.pl

**Keywords:** *Gentiana capitata*, isosaponarin, PC-12 cell line, cell viability, secondary metabolites, HPLC method validation

## Abstract

Some species of the Gentianaceae family are a valuable source of secondary metabolites. However, the phytochemical knowledge of some of these species remains insufficient. Therefore, this work focused on the isolation of the two main secondary metabolites in the methanolic extract from a *Gentiana capitata* cell suspension using preparative HPLC and the determination of their structure using UHPLC–DAD–IT–MS/MS and NMR methods. Their content in the methanolic extract was quantified using a previously validated HPLC method. The toxicity of the extract and two isolated compounds was also tested on the PC-12 cell line. The structures of the main secondary metabolites were identified as isosaponarin and 3,7,8-Trimethoxy-9-oxo-9H-xanthen-1-yl 6-*O*-*β*-D-ribopyranosyl-*β*-D-allopyranoside by comparing the UHPLC–DAD–IT–MS/MS and NMR results with the literature data. The content of isosaponarin was determined to be 0.76 ± 0.04%, and the content of 3,7,8-trimethoxy-9-oxo-9H-xanthen-1-yl 6-*O*-*β*-D-ribopyranosyl-*β*-D-allopyranoside was found to be 0.31 ± 0.02% in the dry extract. Additionally, a two-fold increase in the viability of the PC-12 cell line was observed compared to the control after treatment with the methanolic extract at a concentration of 500 µg/mL. These results suggest the potential use of *G. capitata* cell suspension methanolic extract as a new source of isosaponarin and 3,7,8-trimethoxy-9-oxo-9H-xanthen-1-yl 6-*O*-*β*-D-ribopyranosyl-*β*-D-allopyranoside, highlighting their lack of toxicity to the PC-12 (rat pheochromocytoma) cell line.

## 1. Introduction

The Gentianaceae Juss. family, known for its diverse range of secondary metabolites such as flavonoids and xanthones, has been extensively studied for its medicinal properties. Various species within this family have been successfully cultivated using cell suspension cultures to enhance the production of these valuable compounds, including the following species: *Swertia minor* (Griseb.) Knobl (swertiamarin) [1], *Gentiana davidi* var. *formosana* (Hayata) T. N. Ho (gentiopicroside, swertiamarin) [2], *Centaurium erythraea* Rafn, and *Centaurium littorale* (Turner) Gilmour (1,5-dihydroxy-3-methoxyxanthon) [3]. Given the successes observed with other members of the Gentianaceae family, employing cell suspension cultures of *Gentiana capitata* Buch.–Ham. ex D.Don is a promising approach to efficiently produce its secondary metabolites. This method not only provides a sustainable source of valuable compounds but also facilitates the detailed study of their structures and biological activities, paving the way for potential pharmaceutical applications.

*Gentiana capitata* Buch.–Ham. ex D.Don is an annual herb endemic to the southern and southeastern regions of the Himalayas [4]. Using biotechnological methods, it was possible to obtain in vitro *G. capitata* cell suspensions characterized by stability and rapid growth. An efficient method of cryopreservation of plant biomass was also developed [4]. However, only one study has examined the secondary metabolites of *G. capitata* cell suspension, and the study failed to precisely determine the structure of the main metabolites in the methanolic extract [4]. Due to the analytical method used—mass spectrometry—it was only possible to assign the metabolites to groups such as flavonoids or xanthones. Flavonoids are important secondary metabolites widely distributed in plants and protect plants from various biotic and abiotic stresses [5]. In plants, glycosylation of flavonoids is considered to play a crucial role in changing their physiological properties, including solubility and bioavailability [6]. Among others, isosaponarin, one of the flavone glycosides, was first isolated from wasabi leaves, its primary and currently used source. However, new sources of isosaponarin still need to be sought due to the problematic cultivation of wasabi. So far, in the Gentianaceae family, which *Gentiana capitata* belongs to, isosaponarin has been detected in the roots of *Gentiana triflora* Pall. [7] and *Gentiana linearis* Froel. [8]. It was also determined from the decoctions of *Gentiana triflora* herbs [9].

Isosaponarin has many biological activities, including increasing collagen synthesis caused by up-regulated TGF-*β* type II receptor (T*β*R-II) and prolyl 4-hydroxylase (P4H) protein production [10]. It also induces hair growth [11] and inhibits the release of glutamic acid from cells, which causes its anti-excitotoxic effects [12]. Unfortunately, the oral bioavailability of isosaponarin is very low, and it is metabolized into isovitexin in the digestive system [13]. Due to this metabolism of isosaponarin and the confirmed effect on the increase in collagen synthesis, the optimal pharmacological use of isosaponarin is topical application.

Another group of secondary metabolites isolated from the Gentianaceae family are xanthones. Most of them are simple xanthones or glycosidic conjugates with corresponding sugars. Xanthones isolated from plant biomass are *C*- or *O*-glycosides. They are a group of chemical compounds with diverse biological activities (neuroprotective, anti-bacterial, anti-carcinogenic, anti-oxidant, and anti-diabetic) [14,15,16,17,18]. The following *C*-glycosides have been isolated from plants of the Gentianaceae family: swertipunicosid [14], 3-*O*-demethylswertipunicosid [15], puniceaside D and puniceaside E from *Swertia punicea* Hemsl [16], and 3,5,6,8-tetrahydroxyxanthone-1-*C*-*β*-D-glucoside from *Swertia mussotii* Franch [17]. Of the above compounds, 3-*O*-demethylswertipunicosid has been shown to have potent neuroprotective activity against H_2_O_2_-induced PC-12 cell damage [16]. Glycosides’ more significant biological activity than aglycones was also demonstrated [17]. Xanthones *O*-glycosides with effects on the central nervous system have been isolated from the following species of the Gentianaceae family: puniceaside B and swertiabisxanthone-I 8′-*O*-*β*-D-glucopyranoside from *Swertia punicea,* which were evaluated for their potential neuroprotective activities against H_2_O_2_-induced PC-12 cell damage using an MTT assay and displayed potent neuroprotective activity [16]; corymbiferin 3-*O*-*β*-D-glucopyranoside and swertiabisxanthone-I 8′-*O*-*β*-D-glucopyranoside from *Gentianella amarella* subsp. *acuta* (Michx.) J.M.Gillett, which showed a weak inhibitory effect on acetylcholinesterase (AChE) and monoamine oxidases (MAO) A and B, which are associated with Alzheimer’s disease [18].

The methanolic extracts from the herbs and roots of plants from the Gentianaceae family are rich in flavonoids and xanthones, but they are a poor source of compounds due to their growth rate. For this reason, this work focused on the isolation, structure determination, and quantification of the main secondary metabolites in the methanolic extract from cell aggregates of *G. capitata* suspension, whose growth kinetics are much faster. Due to previous studies that showed the influence of xanthones isolated from the Gentianaceae family on nerve cells, it was also decided to check the influence of the main metabolites found in the methanolic extract and the extract itself on the viability of the rat pheochromocytoma PC-12 cell line.

Cell viability tests of the PC-12 line using the MTT assay are most often performed in one of three types of culture medium in which the compounds with which the cells are treated are dissolved. The first is the medium with typical (normal) FBS (fetal bovine serum) content; tested compounds are dissolved in the same medium where cells are grown and seeded; the second is the medium with reduced FBS content (around 2% FBS), and the third is the medium without FBS. Therefore, these media differ in the content of FBS, which also results in different contents of proteins that may, by binding to compounds, influence the amount of the free fraction of the tested molecule available to cells, which can alter their impact on cell viability. Media also differ in their content of hormones such as insulin or thyroid hormones. Thyroid hormones, in turn, influence the cell’s metabolic rate, which may impact the measured mitochondrial metabolic activity in the case of the MTT assay. The FBS also contains compounds with mitogenic-like effects, which, in the case of the MTT assay, may result in an increase in the number of cells and, therefore, in a higher measured metabolic activity of mitochondria.

For these reasons, the aim of the study was to isolate the main secondary metabolites in the methanolic extract from the *G. capitata* cell suspension, determine their chemical structure using the UHPLC–DAD–IT–MS/MS and NMR methods, and quantify their content through the validated HPLC method. Due to reports on the potential effect of secondary metabolites isolated from the Gentianaceae family, mainly xanthones and some flavonoids, on nerve cells, the aim of this study was also to investigate the viability of the PC-12 cell line after exposure to methanolic extract and the main isolated compounds under conditions of typical (normal) and reduced FBS content.

## 2. Results

### 2.1. Preparative HPLC Purification

**Figure 1:**
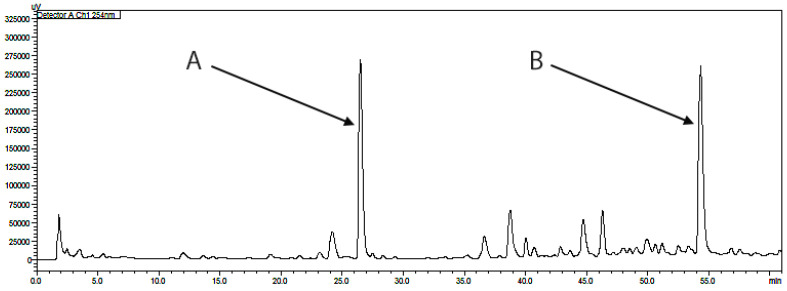
Preparative HPLC–UV chromatogram at 254 nm obtained during separation of *Gentiana capitata* methanolic extract. The collected fractions have been marked as follows: A—compound 2; B—compound 1.

During isolation, two fractions were obtained, frozen and then lyophilized, thus obtaining 36 mg of compound 2 (fraction A) and 17 mg of compound 1 (fraction B) (Figure 1). The UV purity (240 nm) determined by the HPLC–DAD of the isolated compounds was ≥95%.

### 2.2. Structure Elucidation

#### 2.2.1. UHPLC–DAD–IT–MS/MS

**Figure 2:**
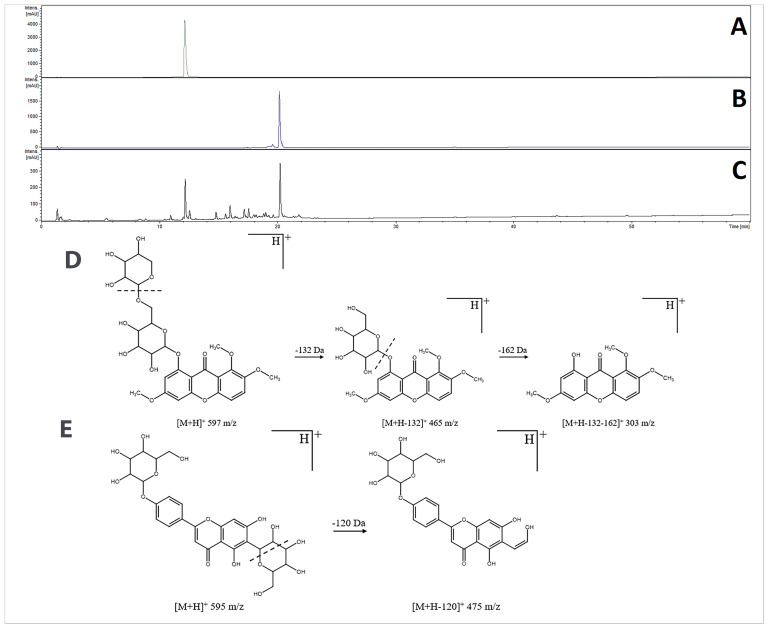
UHPLC–DAD–IT–MS/MS chromatograms at 240 nm. Compounds isolated on preparative HPLC: compound 2 (**A**), compound 1 (**B**) and methanolic extract from *G. capitata* aggregates (**C**), fragmentation pattern of Compound 1 (**D**), and Compound 2 (**E**). The dashed line indicates where bonds break in the ion trap.

The UV chromatograms of isolated compounds obtained at 240 nm show that they are pure enough to be treated as standards for validation (Figure 2A,B).

Compound 1—signal ESI+ 597 *m*/*z* [M+H]^+^ and ESI− 641 *m*/*z* [M+HCOO]^−^. Fragmenting in MS2- to 595 with a loss of 46 *m*/*z* (loss of formic acid HCOOH) indicates the compound mass is 596 amu. Fragmentation of 597 *m*/*z* in MS2+ to 465 and 303 with a loss of 132 and 162 *m*/*z*, respectively, suggests the presence of *O*-pentoside and *O*-hexoside in the molecule (Figure 1).

Compound 2—signal ESI+ 595 *m*/*z* [M+H]^+^ and ESI- 593 *m*/*z* [M−H]^−^ indicates the compound mass is 594 amu. Fragmentation of 595 in MS2+ to 475 and 593 in MS2- to 473 with a loss of 120 *m*/*z* indicates the presence of *C*-glycoside in the molecule (Figure 1).

#### 2.2.2. NMR

The raw 1D and 2D NMR data for compound 1 were transferred and processed in an ACD/Spectrus Processor 2020.1.1 NMR Workbook Suite (Advanced Chemistry Development, Inc. (ACD/Labs), Toronto, ON, Canada, www.acdlabs.com). Then, to find the structure of an unknown natural substance, we used the ACD/Structure Elucidator (ACD/SE) Suite tool [19], which is an expert system for automated structure elucidation of small molecule structures from solution-state NMR data. The molecular formula (MF) was determined from the monoisotopic mass given after analysis by mass spectrometry and later confirmed by analysis of the NMR data, which consisted of 1H, 13C, 1H–13C HSQC, 1H–13C HMBC, and 1H COSY. A total of 197109 structures have been generated by the Correlation Spectroscopy Based Generator, and 484 structures have been stored. The best structure was indicated by the deviation between experimental and predicted chemical shifts using the artificial neural networks predictions approach (dN(13C + 1H) factor) = 5.089 (for second and third dN(13C + 1H), the factors were 5.103 and 5.383, respectively), and deviation between experimental and predicted chemical shifts using the HOSE code approach (dA(13C + 1H) factor) = 1.359 (for second and third dA (13C + 1H), the factors were 1.751 and 2.144, respectively).

**Figure 3:**
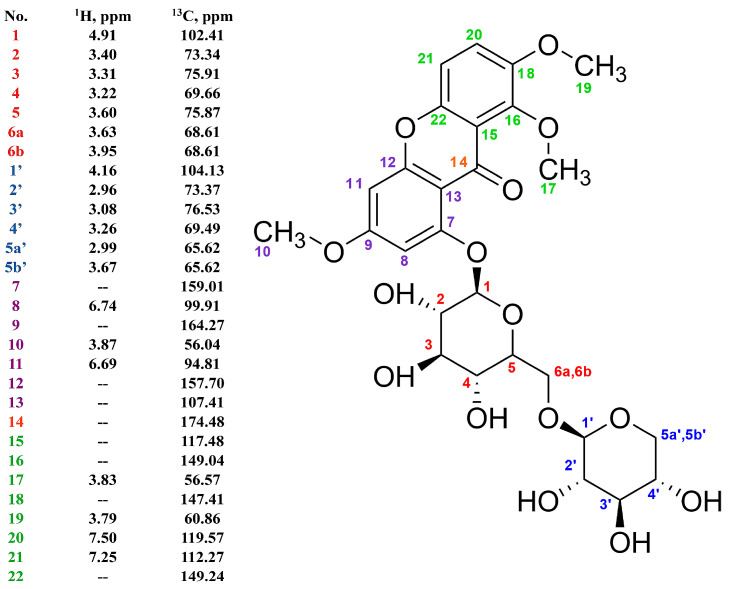
Structure of compound 1.

Additionally, the auto-assignment tool manually verified the proposed assignment with Match Factor (MF) = 0.92 as well (in Mnova 12.0 software (Mestrelab Research., Spain)). The entire structure elucidation report and the elucidation protocol from the ACD/SE Suite tool are in Supporting Information. The determined structure of the chemical compound is shown in Figure 3.

The row 1D and 2D NMR data for isosaponarin were also transferred and processed in an ACD/Spectrus Processor 2020.1.1 NMR Workbook Suite. Then, to find the structure of an unknown natural substance, we used the ACD/Structure Elucidator (ACD/SE) Suite tool. The molecular formula (MF) was determined from the monoisotopic mass given after analysis by mass spectrometry and later confirmed by analysis of NMR data, which consisted of 1H, 13C, 1H–13C HSQC, 1H–13C HMBC, and 1H COSY. After app. 122 h of the elucidation process, the 779319 structures had been generated by the Correlation Spectroscopy Based Generator, and 430 structures had been stored. The best structure was indicated by the deviation between experimental and predicted chemical shifts using the artificial neural networks predictions approach (dN(13C + 1H) factor) = 3.779 (for second and third dN(13C + 1H), the factors were 4.341 and 4.889, respectively, after removal of repeating structures) and deviation between experimental and predicted chemical shifts using the HOSE code approach (dA(13C + 1H) factor) = 0.664 (for second and third dA(13C + 1H), the factors were 1.087 and 1.566, respectively, after removal of repeating structures).

**Figure 4:**
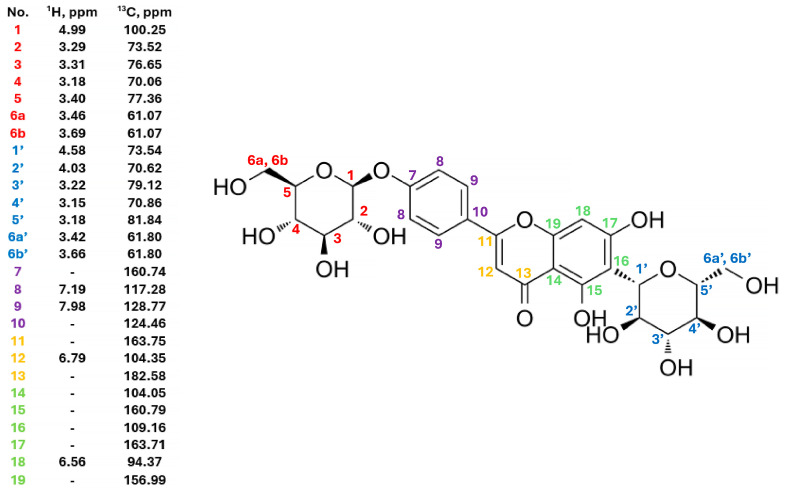
Structure of compound 2.

Additionally, the auto-assignment tool manually verified the proposed assignment with Match Factor (MF) = 0.87 as well (in Mnova 12.0 software). The entire structure elucidation report and the elucidation protocol from the ACD/SE Suite tool are in Supporting Information. The determined structure of the chemical compound is shown in Figure 4.

### 2.3. HPLC Method Validation and Quantification

**Figure 5:**
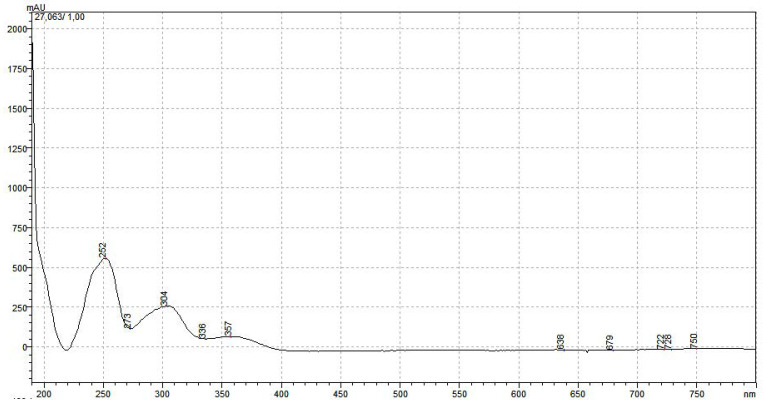
UV–Vis spectrum of compound 1.

**Figure 6:**
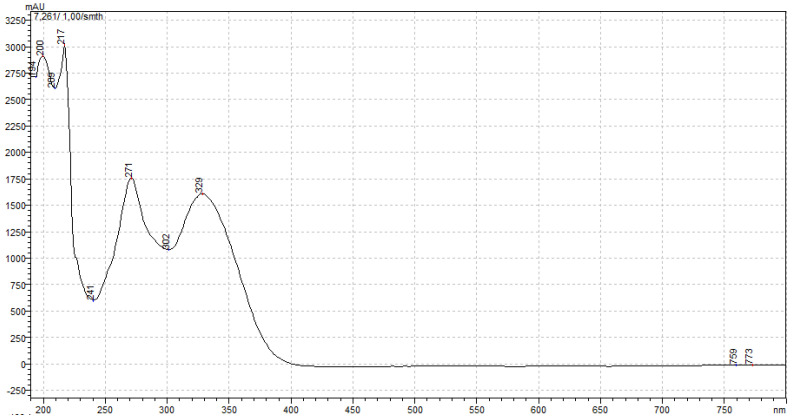
UV–Vis spectrum of compound 2.

**Figure 7:**
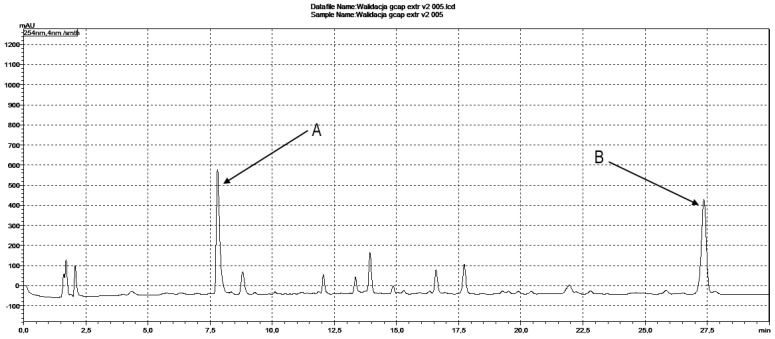
HPLC chromatogram of *Gentiana capitata* cell suspension aggregates methanolic extract at 254 nm; peaks have been marked as follows: A—compound 2; B—compound 1.

**Figure 8:**
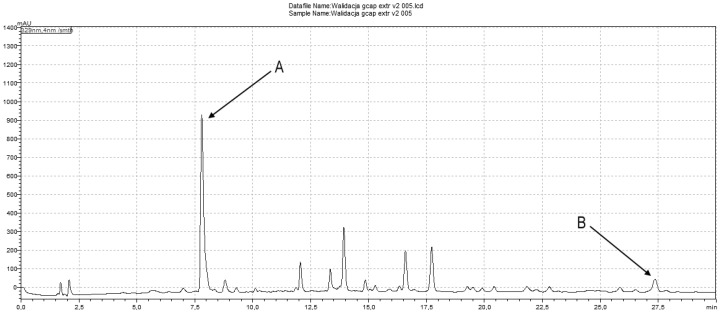
HPLC chromatogram of *Gentiana capitata* cell suspension aggregates methanolic extract at 329 nm; peaks have been marked as follows: A—compound 2; B—compound 1.

Compound 1 and compound 2 were quantified at 254 and 329 nm, respectively, according to their UV–VIS spectra (Figure 5 and Figure 6). Their retention times in analysis were 26.73 ± 0.11 and 7.24 ± 0.03 min (Figure 7 and Figure 8). The parameters of validation were calculated and presented in Table 1.

Quantification of the extract showed a 0.31 ± 0.02% and 0.76 ± 0.04% content of pure compound 1 and compound 2, respectively, in the dry methanolic extract of *G. capitata* aggregates. The content of pure compound 1 and compound 2 in the dry biomass of *G. capitata* was 0.10 ± 0.01% and 0.23 ± 0.01%, respectively.

### 2.4. Biological Activity

**Figure 9:**
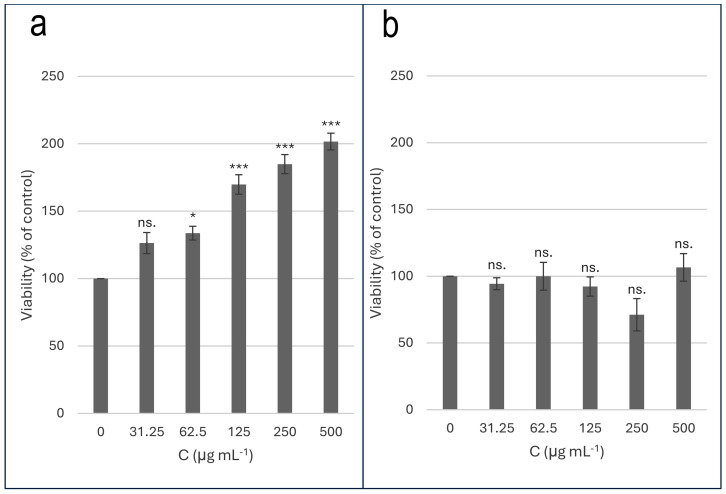
Toxicity of *G. capitata* cell suspension aggregates methanolic extract on PC-12 cell line; (**a**)—low-serum conditions; (**b**)—normal-serum conditions. The data were expressed as mean ± SEM; ns.—non-significant; * *p* < 0.05; ** *p* < 0.01; *** *p* < 0.001 as compared to control, tested with post hoc HSD Tukey’s test.

The methanolic extracts of the *G. capitata* aggregates did affect cell viability in low-serum conditions. They did not exert a statistically significant cytotoxic effect on the PC-12 cells concerning control in any conditions. In the low-serum conditions, for a concentration of 62.5 µg/mL (*p* < 0.05) and concentrations of 125, 250, and 500 µg/mL (*p* < 0.001), a statistically significant increase in cell viability was demonstrated compared to the control with a maximum rise of 200% for a concentration of 500 µg/mL. There were no statistically significant differences in cell viability in the normal-serum conditions. The results are presented in charts (Figure 9).

**Figure 10:**
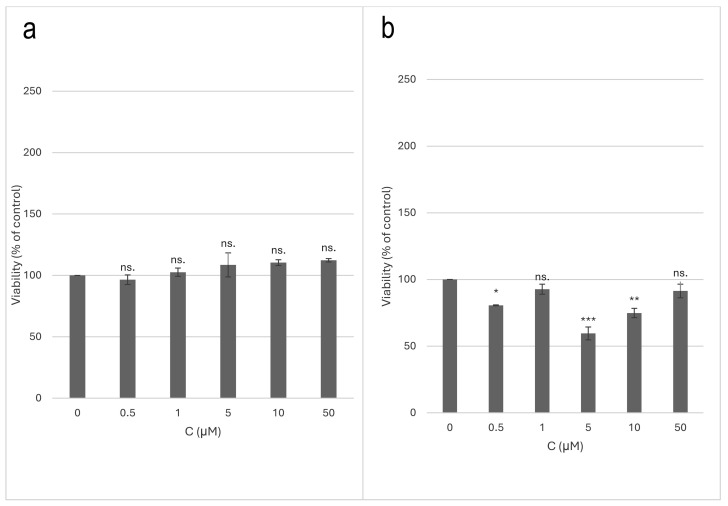
Toxicity of compound 1 on PC-12 cell line; (**a**)—low-serum conditions; (**b**)—normal-serum conditions. The data were expressed as mean ± SEM; ns.—non-significant; * *p* < 0.05; ** *p* < 0.01; *** *p* < 0.001 as compared to control, tested with post hoc HSD Tukey’s test.

Compound 1 did not affect cell viability in low-serum conditions but did exert a cytotoxic effect on the PC-12 cells concerning control in normal-serum conditions. There were no statistically significant differences in cell viability in the low-serum conditions. In the normal-serum conditions, for a concentration of 0.5 µM (*p* < 0.05) and concentrations of 5 µM (*p* < 0.001) and 10 µM (*p* < 0.01), a statistically significant decrease in the cell viability was demonstrated compared to the control. The results are presented in charts (Figure 10).

**Figure 11:**
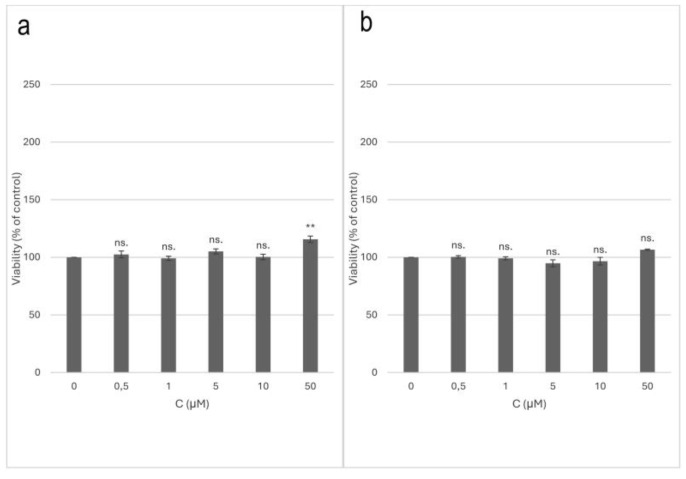
Toxicity of compound 2 (isosaponarin) on PC-12 cell line; (**a**)—low-serum conditions; (**b**)—normal-serum conditions. The data were expressed as mean ± SEM; ns.—non-significant; * *p* < 0.05; ** *p* < 0.01; *** *p* < 0.001 as compared to control, tested with post hoc HSD Tukey’s test.

Compound 2 (isosaponarin) did affect cell viability in low-serum conditions but did not exert a statistically significant cytotoxic effect on the PC-12 cells concerning control in any conditions. In the low-serum conditions, for a concentration of 50 µM (*p* < 0.01), a statistically significant increase in the cell viability was demonstrated compared to the control. There were no statistically significant differences in the cell viability in the normal-serum conditions. The results are presented in charts (Figure 11).

The equivalent of compound 1 and compound 2 in the extract, calculated based on a validated quantification method, does not correspond to the differences in the viability of the PC-12 cell line between treatment with the extract and specific isolated chemical compounds (Table 2).

## 3. Discussion

The isolation of secondary metabolites from plant biomass currently plays a very important role in searching for lead structures for new drugs [20,21]. So far, only a few papers have been published on the phytochemistry of cell suspensions, including only one on the phytochemistry of *Gentiana capitata* species [2]. In a previous work, the authors determined the profile of secondary metabolites of methanolic extract from *G. capitata* suspension culture aggregates using the UHPLC–DAD–IT–MS/MS method [4]. However, based on these results, it was impossible to precisely determine the chemical structure of the main metabolites due to the specificity of the analytical method UHPLC–DAD–IT–MS/MS, which does not allow for determining which isomers were detected.

Isolation of plant secondary metabolites is a multi-stage and time-consuming process [22,23,24]. Individual chemical compounds are isolated from specific fractions, the structure of which is determined by analyzing spectra obtained by mass spectrometry. Based on the spectrum, it is possible to determine the mass of the compound and analyze characteristic fragmentation patterns. It is also possible to determine the structure of aglycone and sugar. Despite a lot of information about the structure provided by the mass spectrum, its limitation is the inability to determine the relative position of the substituents in the molecule. Nuclear magnetic resonance (NMR) spectrometry determines the position of substituents relative to each other. However, it is necessary to isolate the chemical compound of sufficient purity, which is difficult to achieve in the case of isolation from a complex matrix of plant biomass. For this reason, in this work, the isolation was limited to the two compounds with the highest content in the methanolic extract.

So far, most scientific publications on plants from Gentianaceae have focused on the metabolism of herbs and roots. In recent years, nearly 600 compounds have been isolated from *Gentiana* species, and some of them have been identified for 20 different biological activities, for example, immunomodulation and hepatic, gastrointestinal, cardiovascular, skin, pulmonary, joint, bone, and reproductive protection [25]. These experiments have focused on cytoprotective effects. Many works also suggest an influence on neurogenesis [26,27,28]. Still, only a few described the impact of extracts or compounds isolated from Gentianaceae on the viability of neuronal lines, including the rat pheochromocytoma PC-12 line [29].

The present study investigated the effect of the extract and two isolated secondary metabolites on the viability of the PC-12 line in low-serum (2% FBS) and normal-serum (10% FBS, 5% HS) conditions. The effects of the compounds have not been studied under serum-free conditions because the serum-deprivation state induces oxidative injury, leading to ATP depletion, apoptosis cascade, and neurodegeneration [30]. The aim of the current experiment was to test the toxicity of the extract and their isolated constituent on neuronal cells, which can be treated in similar conditions to healthy organisms in vivo. In the case of normal serum conditions, i.e., conditions in which the cells are cultured in a standard manner, where they can divide due to the presence of a sufficient amount of FBS, no statistically significant differences in cell viability were demonstrated compared to the control (DMSO) for methanolic extract and isosaponarin in every concentration. However, a statistically significant difference occurred during the compound 1 treatment in normal-serum conditions. The viability of PC-12 decreased in a lower range of concentrations during treatment. A decrease in the cell viability was not present during extract treatment, even when the extract contained a compound 1 toxic equivalent. This observation can be explained by the simultaneous effect of other compounds present in the extract, which causes the elimination of toxicity. Different data were obtained during the low-serum conditions, i.e., conditions in which the cells cannot divide due to a too-low FBS content in the medium. The viability doubled in a dose-dependent manner during the extract treatment. Still, this effect cannot be explained by the presence of the main compounds (compound 1 and isosaponarin) in the extract because their impact on the cells in these conditions was negligible. The obtained results suggest that in the extract, there are compounds (or a compound) present, which are potent viability enhancers at lower concentrations, or a synergistic effect is present between the extract constituents. The differences in the extract and isolated secondary metabolites impact the PC-12 cells during low-serum and normal-serum experiments and suggest that metabolites can bind to proteins present in FBS in normal-serum conditions. It may explain the differences in PC-12 cell viability between low serum and normal serum on extract treatment. Another explanation is that the compounds can bind to cell receptors and act as allosteric modulators for signal molecules present in the FBS. That can explain the lack of action of compound 1 in low-serum conditions but decreased viability in normal-serum conditions. Further study should be concentrated on exploring these phenomena.

## 4. Materials and Methods

### 4.1. Chemicals and Reagents

An Acetonitrile HPLC grade, Methanol HPLC grade Chromasolv ^®^, Formic acid, and HPLC grade water were purchased from Merck KGaA. An LC–MS H_2_O (18 Ω) grade was purchased from Millipore (Burlington, MA, USA). An LC–MS Acetonitrile grade was purchased from Merck (Darmstadt, Germany). Sucrose was purchased from POCH, Gliwice, Poland. A dichlorophenoxyacetic acid (2,4-D) and kinetin were purchased from Grand Island Biological Company, New York, NY, USA. A mixture of Murashige and Skoog (MS) salts and vitamins was purchased from Duchefa Biochemie, The Netherlands. Dimethyl sulfoxide (DMSO); 3-(4,5-dimethylthiazol-2-yl)-2,5-diphenyltetrazolium bromide (MTT); poly-*D*-lysine hydrobromide 30-70 kDa (PDL); Dulbecco’s modified Eagle’s medium—high glucose 4500 mg/L glucose (DMEM); fetal bovine serum heat inactivated (FBS), and horse serum (HS) were purchased from Merck KGaA. L-glutamine (200 mM solution) and antibiotic–antimycotic (100X solution) were purchased from ThermoFisher Scientific (Waltham, MA, USA).

### 4.2. Experimental Material and Culture Conditions

**Figure 12:**
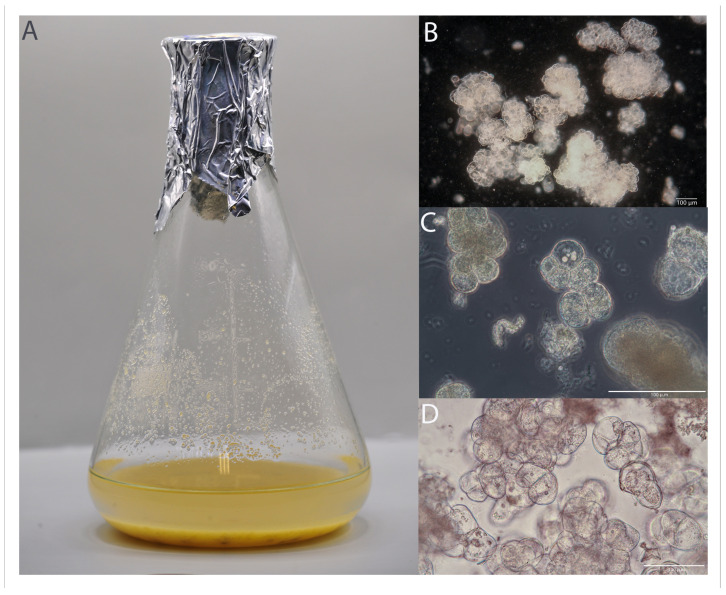
Cell suspension cultures of *G. capitata* on MS-based medium. Cell suspension in an Erlenmeyer flask (**A**) with capacity of 0.5 L and morphology of cell suspensions. Embryogenic suspension cultures developed from cells are shown in dark field (**B**) and bright field (**D**). Multicellular clumps of 3-month-old culture observed by light microscopy with phase contrast (**C**).

The *G. capitata* cell suspension culture (Figure 12) was utilized during our previous research [4]. Seeds obtained from the Botanical Garden in Teplice, Czech Republic, were utilized to initiate in vitro cultures. Prior to the establishment of these cultures, the seeds were surface-sterilized by submerging them in 70% ethyl alcohol for 1 min, followed by a 15-min soak in a 30% ACE (Procter & Gamble) solution (*v*/*v*) in water [4]. The seeds were then rinsed three times with sterile distilled water and were placed on a half-strength Murashige and Skoog (MS) medium [31] with 0.5 mg/L gibberellic acid (GA_3_), 1.5% sucrose (*w*/*v*), and 0.8% agar for germination. The seeds were incubated in a plant-breeding room with diffuse light at an intensity of 100 μmol·m^−2^·s^−1^ and a 16-h photoperiod, maintained at a temperature of 22 ± 1 °C. After germination, cotyledons were excised from the seedlings and transferred to a solid MS medium with full strength, including 1.0 mg/L kinetin (Kin), 0.5 mg/L 2,4-dichlorophenoxyacetic acid (2,4-D), and 3% sucrose (*w*/*v*) to induce callus formation [4]. Cell suspension cultures were generated by transferring callus tissue to liquid MS medium [4]. These cultures were maintained in a growth chamber at 22 ± 1 °C in the dark on a rotary shaker at 100 rpm. After seven days of culture, the medium was replaced with fresh medium. Subsequently, after another seven days during passage, the biomass was divided in a 1:1 (*v*/*v*) ratio. One half was further cultured in 100 mL of medium following the previously described procedure, while the second half was vacuum filtered, and the aggregates were frozen at −20 °C for compound isolation. The flasks (0.5 L) were maintained in the dark at a growth chamber temperature of 23 ± 1°C on Innova 2300 shaking platforms (105 RPM).

### 4.3. Extraction and Isolation

#### 4.3.1. Preparation of the Methanolic Extract

The frozen biomass was lyophilized in Alpha 2-4 LSCplus laboratory freeze dryer. The dry *G. capitata* cell aggregates were finely powdered using a mortar. Next, the ground biomass was subjected to triple extraction with methanol at a 1:10 (*m*/*v*) biomass-to-solvent ratio, followed by a 15 min of sonication. Then, the methanolic extract was evaporated to dryness at 40 °C using a Heidolph Laborota 4000 rotary evaporator, and the dry methanolic extract was dissolved in the DMSO at a 500 mg/mL concentration. The resulting extract was centrifuged in MiniSpin plus at 6000× g for 30 min; the supernatant was filtered through a 5 µm syringe filter and subjected to a preparative HPLC system.

#### 4.3.2. Preparative HPLC

The preparative HPLC compound isolation was performed using an apparatus equipped with a dual low-pressure gradient pump LC-20AP, a sampler SIL-10AF, a CTO-10AS column oven set at 25 °C, a diode array detector SPD-10AVP and a fraction collector FRC-10A (Shimadzu, Kyoto, Japan). Isolation was carried out on a reversed-phase Kinetex XB-C_18_ column (150 × 21.2 mm, 5 µm; Phenomenex, Torrance, CA, USA). The mobile phase (A) was 0.1% formic acid in water (*v*/*v*), and the mobile phase (B) was 0.1% formic acid in acetonitrile (*v*/*v*). A gradient solvent system separation was used as follows: 0–60 min, 2–26% B; 60–61 min, 95% B. The flow rate was 20.0 mL/min. The volume of injected samples was 400 µL. Elution was monitored at 254 nm. LabSolutions system (Shimadzu, Kyoto, Japan) was used to manage operating procedures and calculations. Collected fractions were frozen and freeze-dried. Isolated compounds were stored at −20 °C.

Multiple chromatographic conditions were tested, and a linear gradient of 2–26% in 60 min was required to separate the dominant compounds in the tested extract and allow the collection of purified compounds. Dry methanolic extract (4.0 g) dissolved in DMSO at 500 mg/mL concentration with multiple 400 µL injections was subjected to a preparative HPLC system to obtain isosaponarin (26–27 min) and xanthone derivative (54–55 min).

### 4.4. Structure Elucidation

#### 4.4.1. UHPLC–DAD–IT–MS

An analysis was performed using an Ultimate 3000 series system (Dionex, Idstein, Germany) equipped with a diode array detector and coupled with an Amazon SL ion trap mass spectrometer (Bruker Daltonik GmbH, Bremen, Germany). The compounds were separated in analyzed samples on a Kinetex XB-C18 column (150 mm × 2.1 mm × 1.9 mm), Phenomenex (Torrance, CA, USA). The column temperature was maintained at 25 °C. The mobile phase (A) was 0.1% formic acid in deionized water (*v*/*v*), and mobile phase (B) was 0.1% formic acid in acetonitrile (*v*/*v*) with a linear gradient as follows: 0 min, 2% B; 60 min, 98% B. The flow rate was 0.3 mL/min. The volume of injected samples was 3 µL of isolated compound dissolved in methanol in 1mg/mL concentration. The eluate was introduced into the mass spectrometer without splitting. The ion trap Amazon SL mass spectrometer was equipped with ESI interface. The parameters for the ESI source were a nebulizer pressure of 40 psi, a dry gas flow of 9 L/min, a dry temperature of 135 °C, and a capillary voltage of 4.5 kV. The compounds were analyzed in positive and negative ion mode. The MS2 fragmentations were performed using Smart Frag mode.

#### 4.4.2. NMR

##### Compound 1

The NMR spectra were acquired at 298 K on an Agilent VNMRS 600 MHz spectrometer, using a 5 mm triple-resonance Triax ((1H, 13C, 15N) x,y,z-PFG probe head) for all experiments, except direct 13C {1H} measurement, for which a 5 mm OneNMR (1H/19F, 15N-31P) z-PFG probe head was used. The sample was dissolved in 0.6 cm^3^ DMSO–d6. The spectrometer frequencies were set to 599.833 MHz and 150.843 MHz for 1H and 13C, respectively. For 1H and 13C, 8.4 and 15.6 ms rectangular p/2 pulses were used, respectively.

The 1D ^1^H spectra were recorded with 16 scans, a relaxation delay of 2 s, and an acquisition time of 1.7 s. In the case of 13C spectra, 4000 scans were accumulated with a relaxation delay of 1 s and an acquisition time of 0.87 s.

The standard VNMRJ 4.2 gHSQCAD pulse sequence was used to acquire a ^1^H–^13^C HSQC experiment. A total of 4 scans were acquired for 400 t1 complex data points, using a relaxation delay of 1.5 s and an acquisition time of 150 ms. The indirect dimension spectral width was set at 24.1 kHz; thus, the maximum t1 evolution period of 16.6 ms was achieved. Cosine square apodization was performed with double zero-filling in the both dimensions prior to Fourier transformation.

The standard VNMRJ 4.2 gHMBCAD pulse sequence was used to acquire the ^1^H–^13^C HMBC experiment. A total of 16 scans were acquired for 200 t1 complex data points, using a relaxation delay of 1.5 s and an acquisition time of 150 ms. The indirect dimension spectral width was set at 31.5 kHz; thus, the maximum t1 evolution period of 12.7 ms was achieved. Cosine square and sine square apodization were performed in the t1 and t2 dimensions, respectively. Double zero-filling in both dimensions was applied prior to Fourier transformation.

The standard VNMRJ 4.2 gCOSY pulse sequence was used to acquire the ^1^H COSY experiment. A total of 8 scans were acquired for 400 t1 real data points, using a relaxation delay of 1.5 s and an acquisition time of 150 ms. The spectral width was set to 4.7 kHz in both dimensions, and the maximum t1 evolution period of 42.7 ms was achieved. Sine square apodization was performed in the both dimensions; zero-filling in the both dimensions to 1024 real points was applied prior to Fourier transformation, and absolute value spectra were calculated.

##### Compound 2

The NMR spectra were acquired at 298 K on an Agilent VNMRS 600 MHz spectrometer, using a 5 mm triple-resonance Triax ((1H, 13C, 15N) x,y,z-PFG probe head) for all experiments, except direct 13C {1H} measurement, for which a 5 mm OneNMR (1H/19F, 15N-31P) z-PFG probe head was used. The sample was dissolved in 0.6 cm^3^ DMSO–d6. The 10 mL of D_2_O was added to remove the peaks of the OH groups. The spectrometer frequencies were set to 599.833 MHz and 150.843 MHz for 1H and 13C, respectively. For 1H and 13C, 8.4 and 15.6 ms rectangular p/2 pulses were used, respectively.

The 1D ^1^H spectra were recorded with 16 scans, relaxation delay of 2 s, and acquisition time of 1.7 s. In the case of 13C spectra 10000 scans were accumulated with relaxation delay of 1 s and acquisition time of 0.87 s.

The ^1^H–^13^C HSQC experiment was acquired using the standard VNMRJ 4.2 gHSQCAD pulse sequence. A total of 4 scans were acquired for 600 t1 complex data points, using a relaxation delay of 1.5 s and an acquisition time of 150 ms. The indirect dimension spectral width was set to 24.1 kHz; thus, the maximum t1 evolution period of 24.9 ms was achieved. The cosine square apodization was performed with double zero-filling in the both dimensions prior to Fourier transformation.

The ^1^H–^13^C HMBC experiment was acquired using the standard VNMRJ 4.2 gHMBCAD pulse sequence. A total of 16 scans were acquired for 800 t1 complex data points, using a relaxation delay of 1.5 s and an acquisition time of 150 ms. The indirect dimension spectral width of 24.883 kHz was set; thus, the maximum t1 evolution period of 32.1 ms was achieved. The cosine square and sine square apodization were performed in t1 and t2 dimensions, respectively. Double zero-filling in the both dimensions was applied prior to Fourier transformation

The ^1^H COSY experiment was acquired using the standard VNMRJ 4.2 gCOSY pulse sequence. A total of 4 scans were acquired for 1024 t1 real data points, using a relaxation delay of 1.5 s and an acquisition time of 500 ms. The spectral width was set to 3.930 kHz in both dimensions, and the maximum t1 evolution period of 261 ms was achieved. Sine square apodization was performed in the both dimensions; zero-filling in the both dimensions to 4096 real points was applied prior to Fourier transformation, and the absolute value spectra were calculated.

The entire structure elucidation reports and the elucidation protocol from the ACD/SE Suite tool (Advanced Chemistry Development, Inc. (ACD/Labs), Toronto, ON, Canada, www.acdlabs.com) for compounds 1 and 2 are included in the Supplementary Materials.

### 4.5. HPLC Method Quantification

The HPLC–DAD analyses were performed using an apparatus equipped with a dual low-pressure gradient pump LC-10AT, a sampler SIL-20A, a CTO-10AS column oven set at 25 °C, and a diode array detector SPD-M20A (Shimadzu, Kyoto, Japan). HPLC analyses were carried out on a reversed-phase Kinetex XB-C18 column (150 × 2.1 mm, 2.6 µm; Phenomenex, Torrance, CA, USA). The mobile phase (A) was 0.1% formic acid in water (*v*/*v*), and the mobile phase (B) was 0.1% formic acid in acetonitrile (*v*/*v*). A multi-step gradient solvent system separation was used as follows: 0–10 min, 10–20% B; 10–30 min, 20–24% B; 30–31 min, 24–100% B; 31–35 min, 100% B; 35–37.5 min, 10% B. The flow rate was 0.4 mL/min. The volume of injected samples was 3 µL. UV–Vis spectra were recorded over a range of 200–450 nm, and chromatograms were acquired at 254 nm and 329 nm. LabSolutions system (Shimadzu, Kyoto, Japan) was used to manage operating procedures and calculations. The content of compounds in samples was calculated using the regression parameters of the calibration curves.

The lyophilized *G. capitata* aggregates were finely powdered using a mortar. Next, 0.5 g of biomass was subjected to triple extraction with 5 mL of methanol, followed by a 15-min sonication. The methanolic extract was evaporated to dryness at room temperature, dissolved in 5 mL HPLC-grade methanol, and centrifuged at 6000× *g* for 5 min. The supernatant was used for HPLC analysis.

The compound content (%) in the extract was calculated using the following formula: compound content = [(CxV)/M]*100, where C is the concentration of the chemical compound in sample (mg/mL) obtained from the calibration curve, V is the volume of solvent in which the extract was dissolved (mL), and M is the mass of dry extract (mg).

### 4.6. HPLC Method Validation

The method developed was validated according to the International Conference on Harmonisation (ICH) guidelines [32]. It was validated using the methanolic extract from lyophilized cell aggregates [33,34].

Multiple tests were performed to optimize chromatographic conditions. A dual-step gradient was used to achieve good separation of peaks and an analysis time of less than 30 min. The optimal method was 10–20% B in 10 min, followed by 20–24% B in 20 min. At the end, the column was flushed with 100% B for 5 min and equilibrated in 10% B for 2.5 min.

#### 4.6.1. Specificity

Specificity was tested by comparing retention times and the UV spectra of substances in extracts with isolated in this paper compound.

#### 4.6.2. Linearity

Linear relationship between compound signals and concentration of compounds determinations were assessed during calibration curve preparation for five concentrations in 6 injections each. The compound was accurately weighted 1 ± 0.1 mg, dissolved in HPLC grade methanol to obtain 1 mg/mL concentration, and further serially diluted in methanol. The correlation coefficient (R2), y-intercept, slope of the regression line, and residual sum of squares were provided.

#### 4.6.3. Range

The working ranges were defined as ranges between limit of quantification values and the highest compound concentrations from linearity determinations.

#### 4.6.4. Accuracy

The method’s accuracy was assessed using three concentration levels covering the specified range. Accuracy was evaluated by testing the recovery of sample solutions. It was reported as percent recovery at levels 80, 100, and 120% of the known added amounts of the tested analytes in the sample. A sample and standard solution in the determined concentration were mixed in a ratio of 1:1 to obtain the appropriate content of the tested compound. The ratio of the known added concentration of standard compound to the concentration of standard compound calculated based on the calibration curve developed with the HPLC–DAD method was determined and expressed as a percentage of recovery.

#### 4.6.5. Precision

Precision was assessed by injecting the prepared extract six times in one day (intraday) and by intermediate precision, by injecting the prepared extract six times each day for three consecutive days (interday).

#### 4.6.6. Limit of Detection (LOD) and Limit of Quantification (LOQ)

Limits of detection and quantitation were calculated using the following formulas: LOD = 3.3 × δ/S and LOQ = 10 δ/S, respectively (δ—standard deviation of the intercept; S—slope of the calibration curves) [18].

### 4.7. Cell Vviability

#### 4.7.1. Cell Culture

The rat pheochromocytoma PC-12 cell line was kindly gifted by Professor A. Adamczyk (Mossakowski Medical Research Centre, Warsaw, Poland). The cells were cultured in complete medium (DMEM 4.5 g/L glucose, 10% heat-inactivated FBS, 5% heat-inactivated HS, 2mM of L–Glutamine, 100 U/mL of penicillin, 100 µg/mL of streptomycin, and 250 ng/mL of amphotericin B). The cells were maintained at 37 °C in a humidified Binder incubator containing 5% CO_2_ atmosphere [35].

#### 4.7.2. Cell Treatment

Equal PC-12 cell numbers were seeded into 96-well plates coated with 0.1% poly-*D*-lysine (30–70 kDa) at a density of 7 × 10^4^/mL in a complete medium. After 24 h, the growth medium was changed to media containing *G. capitata* methanolic extract (31.25–500 µg/mL) or isolated compounds (0.5–50 µM). The aggregate methanolic extract and isolated compound were primarily dissolved in DMSO and further diluted in complete medium or low serum medium (DMEM 4.5 g/L glucose, 2% heat-inactivated FBS, 2mM of L–Glutamine, 100 U/mL of penicillin, 100 µg/mL of streptomycin, and 250 ng/mL of amphotericin B). The final DMSO concentration in each solution was equal to 0.5% [35].

#### 4.7.3. Determination of Cell Viability

The cell viability was determined with an MTT assay. After 48 h of incubation in the presence of tested compounds, the medium was changed to serum-free DMEM containing MTT (2mM), and the cells were incubated for three hours. Then, the medium was removed, the cells were dissolved in DMSO, and absorbance was recorded at 570 and 630 nm on BioTek Synergy 4. The cell viability was calculated from 570 to 630 nm absorbance difference and expressed as a percentage of control containing only 0.5% DMSO.

## 5. Conclusions

A source and method for the biosynthesis of isosaponarin and, for the first time, the xanthone 3,7,8-trimethoxy-9-oxo-9H-xanthen-1-yl 6-*O*-*β*-D-ribopyranosyl-*β*-D-allopyranoside were successfully developed using *Gentiana capitata* suspension cultures. Additionally, the HPLC–DAD method for determining the content of both compounds was validated. The tests conducted on the extracts and isolated compounds with PC-12 cells indicate that the observed effects are not solely attributable to the isolated compounds but rather to other constituents present within the cell suspension extracts.

## Figures and Tables

**Table 1:** Parameters of validation.

Parameters of Validation		Compound 1		Compound 2	
Linearity	Regression equation	y = 7700.4x + 274673		y = 5269.2x + 39531	
	r	0.9985		0.9999	
	R^2^	0.9971		0.9998	
	test F (α = 0.99)	2.42		2.23	
Recovery (n = 6)		(%)	CV (%)	(%)	CV (%)
	80% of content	116.34	0.78	96.34	0.74
	100% of content	104.58	0.37	91.62	0.56
	120% of content	97.48	0.73	85.02	1.82
Repeatability (n = 6)	mean (ng)	829.14		854.07	
	S (ng)	5.81		10.95	
	CV (%)	0.7		1.28	
	x ± ∆x (α = 0.05) (ng)	829.14 ± 6.09		854.07 ± 11.49	
Intermediate precision (n = 6)	mean (ng)	853.41		858.89	
	S (ng)	30.14		7.77	
	CV (%)	3.53		0.90	
	x ± ∆x (α = 0.05) (ng)	853.41 ± 31.63		858.89 ± 8.15	
LOQ (ng) (n = 6)		229		163	
LOD (ng) (n = 6)		76		54	
Range (ng)		229–1200		163–3000	

CV—coefficient of variation; S—standard deviation; LOQ—limit of quantification (per injection); LOD—limit of detection (per injection).

**Table 2:** Compound 1 and compound 2 equivalent in methanolic extract obtained from *G. capitata* cell suspension aggregates.

Extract Concentration [µg/mL]	Compound 1 Equivalent ± SD [µM]	Compound 2 Equivalent ± SD [µM]
500	2.60 ± 0.14	6.38 ± 0.31
250	1.30 ± 0.07	3.19 ± 0.16
125	0.65 ± 0.03	1.60 ± 0.08
62.5	0.33 ± 0.02	0.80 ± 0.04
31.25	0.16 ± 0.01	0.40 ± 0.02
0	0	0

## Data Availability

The data presented in this study are available on request from the corresponding author.

## Supplementary Materials

The following supporting information can be downloaded at: https://www.mdpi.com/article/10.3390/ijms25168576/s1.
