# Supplementary material for: Gentiana capitata Buch.–Ham. ex D.Don Cell Suspension Culture as a New Source of Isosaponarin and 3,7,8-Trimethoxy-9-oxo-9H-xanthen-1-yl 6-O-β-D-ribopyranosyl-β-D-allopyranoside and Their Effect on PC-12 Cell Viability
Source: Int J Mol Sci. 2024 Aug 6;25(16):8576. doi: 10.3390/ijms25168576 (PMC11354924; doi:10.3390/ijms25168576)
Supplement: Supplementary file 1 [file ijms-25-08576-s001.zip › Structure Elucidation Report for Compound 1.pdf]

The entire structure elucidation report and the elucidation protocol from the ACD/SE Suite tool (Advanced Chemistry Development, Inc. (ACD/Labs), Toronto, ON, Canada, www.acdlabs.com) for **compound 1**

Structure Elucidation Report for compound 1

Initial Data

Composition Restrictions:
Molecular Weight = 0.000-1000.000
Double Bonds Equivalent = 0.00-100.00
Allowed Composition = C(0-100) H(0-100) O(0-20) N(0-10)
Molecular Formula = C27H32O15

Spectral Data:
standard 1H - 20 peaks
merged 1H - 20 peaks
standard 13C - 27 peaks
merged 13C - 27 peaks
COSY 1H-1H - 25 peaks
HSQC-DEPT 13C-1H - 20 peaks
HMBC 13C-1H - 49 peaks

Most Probable Structure

Following structure has been placed to the first position after spectra calculation

Structure Elucidation Report for compound 1 (Page 2)

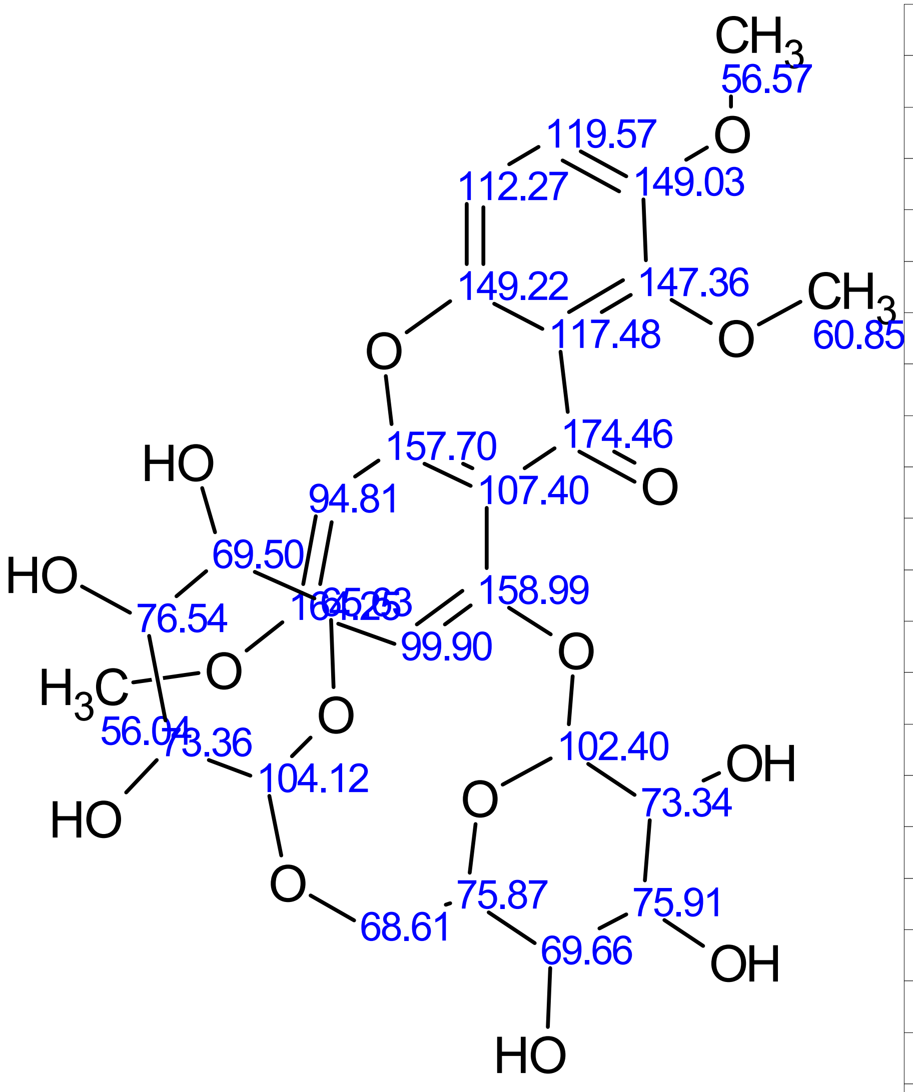

Carbon Assignment

#	N	Shift (ppm)	Atoms	XHn
1	1	56.035	1	CH3(q)
2	2	56.568	1	CH3(q)
3	3	60.848	1	CH3(q)
4	4	65.632	1	CH2(t)
5	5	68.609	1	CH2(t)
6	6	69.496	1	CH(d)
7	7	69.659	1	CH(d)
8	8	73.343	1	CH(d)
9	9	73.356	1	CH(d)
10	10	75.868	1	CH(d)
11	11	75.906	1	CH(d)
12	12	76.542	1	CH(d)
13	13	94.806	1	CH(d)
14	14	99.900	1	CH(d)
15	15	102.398	1	CH(d)
16	16	104.123	1	CH(d)
17	19	107.405	1	C(s)
18	17	112.269	1	CH(d)
19	20	117.476	1	C(s)
20	18	119.566	1	CH(d)
21	21	147.359	1	C(s)
22	22	149.026	1	C(s)
23	23	149.216	1	C(s)
24	24	157.699	1	C(s)
25	25	158.994	1	C(s)
26	26	164.253	1	C(s)

Structure Elucidation Report for compound 1" (Page 3)

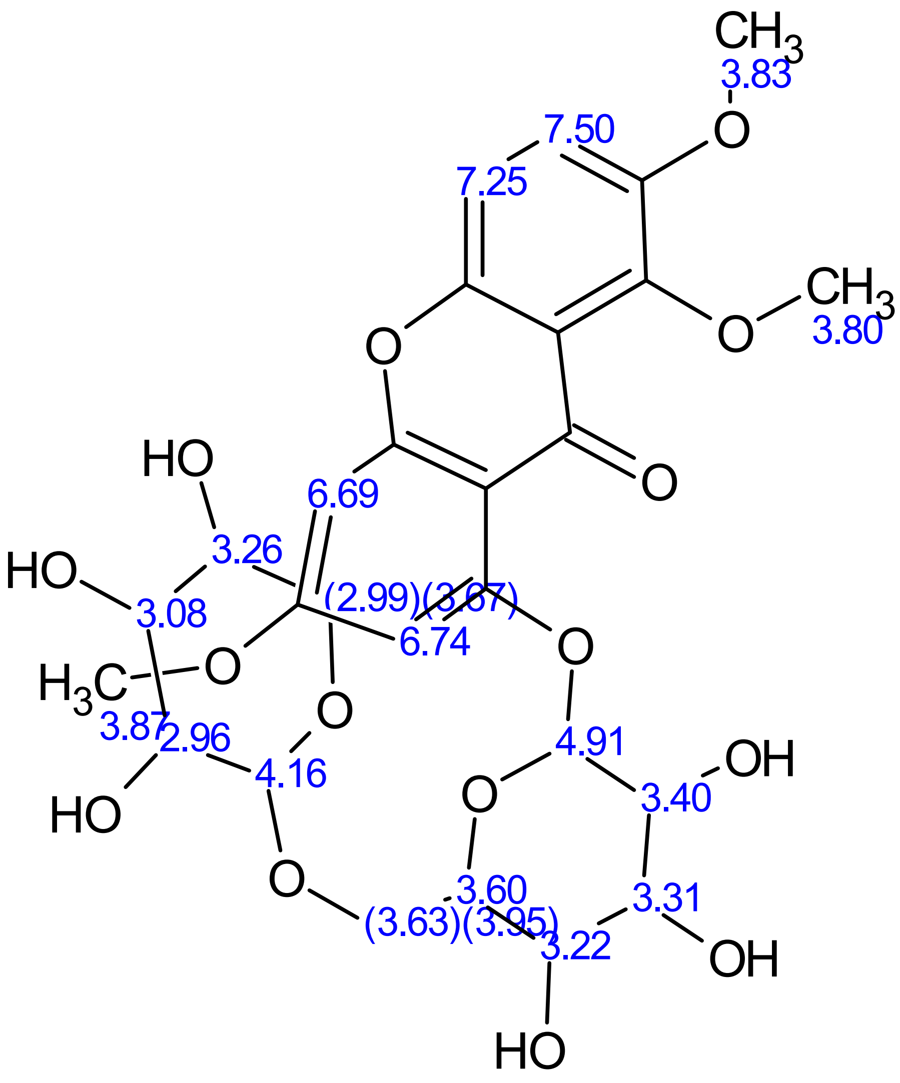

Proton Assignment

#	N	Mark	Shift (ppm)	Protons
1	9		2.959	1
2	4		2.987	1
3	12		3.077	1
4	7		3.220	1
5	6		3.256	1
6	11		3.312	1
7	8		3.404	1
8	10		3.600	1
9	5		3.627	1
10	4		3.666	1
11	3		3.795	3
12	2		3.833	3
13	1		3.869	3
14	5		3.950	1
15	16		4.161	1
16	15		4.911	1
17	13		6.694	1
18	14		6.744	1
19	17		7.247	1

Generated Structures

1 (ID=425)<br>The Best Structure<br>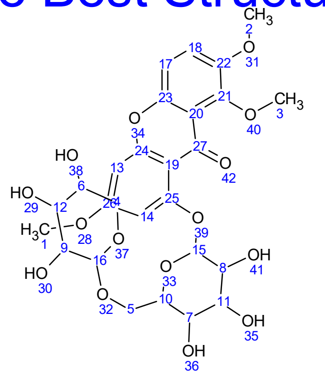	2 (ID=418)<br>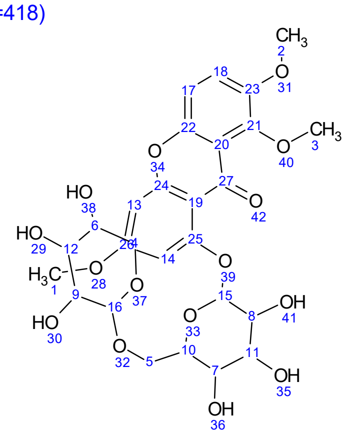	3 (ID=426)<br>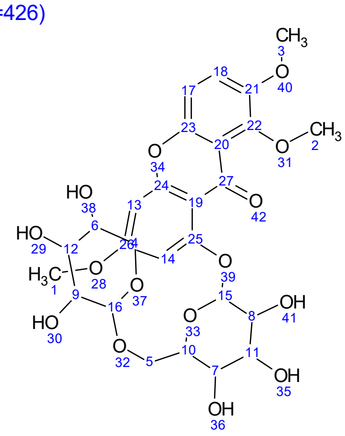
$d_N(^{13}C+^{1}H)$: 5.089	$d_N(^{13}C+^{1}H)$: 5.103	$d_N(^{13}C+^{1}H)$: 5.383
4 (ID=378)<br>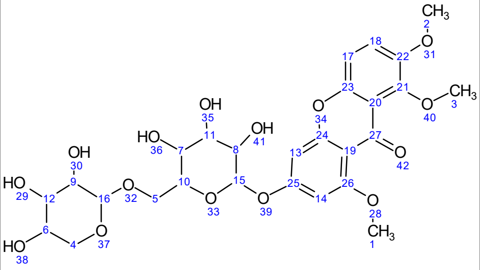	5 (ID=371)<br>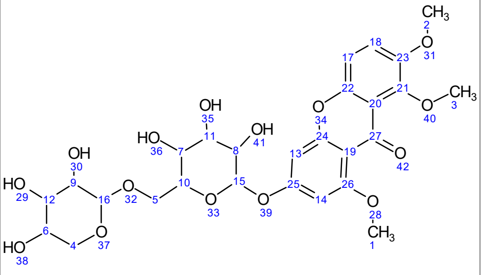	6 (ID=420)<br>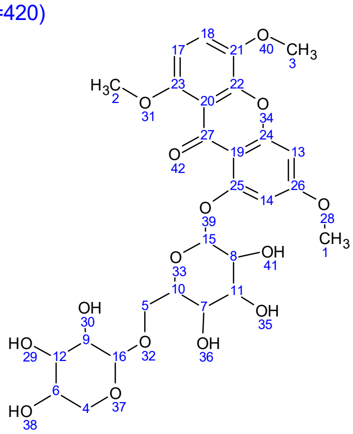
$d_N(^{13}C+^{1}H)$: 5.476	$d_N(^{13}C+^{1}H)$: 5.490	$d_N(^{13}C+^{1}H)$: 5.725

Structure Elucidation Report for compound 1 (Page 5)

7 (ID=379)

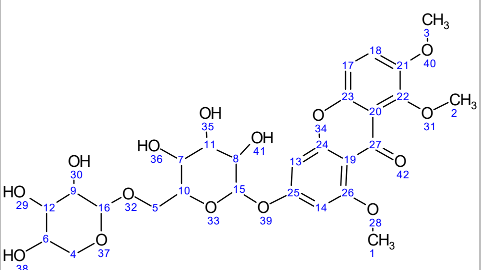

$d_N(^{13}C+^{1}H)$: 5.770

8 (ID=419)

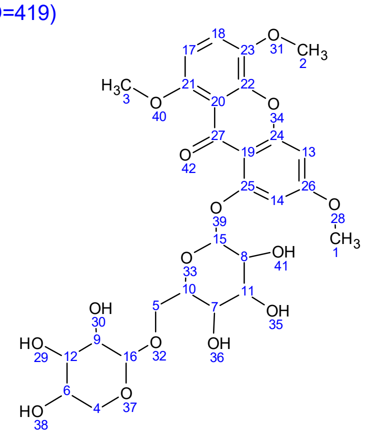

$d_N(^{13}C+^{1}H)$: 5.863

9 (ID=417)

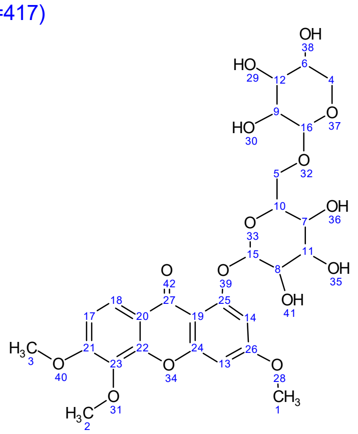

$d_N(^{13}C+^{1}H)$: 5.903

10 (ID=373)

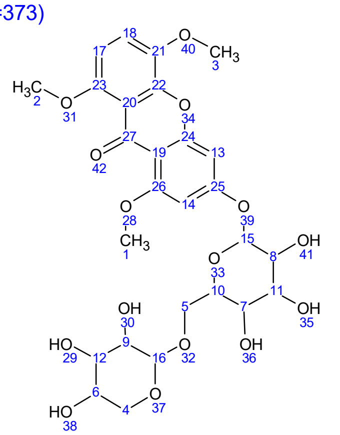

$d_N(^{13}C+^{1}H)$: 6.134

11 (ID=372)

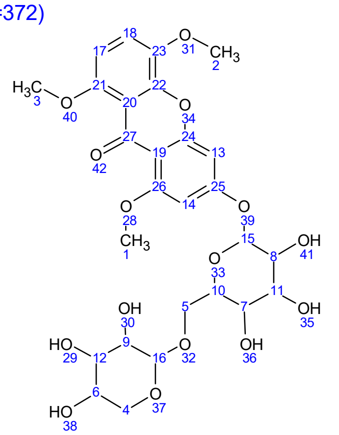

$d_N(^{13}C+^{1}H)$: 6.271

12 (ID=421)

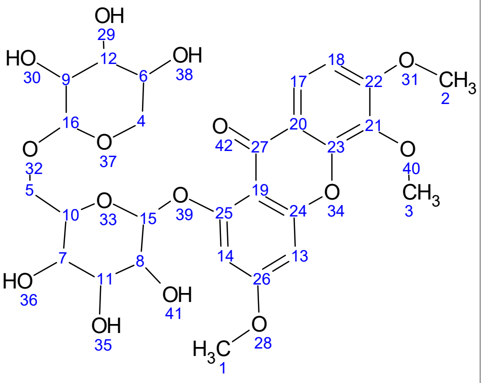

$d_N(^{13}C+^{1}H)$: 6.286

Structure Elucidation Report for compound 1 (Page 6)

13 (ID=370)	14 (ID=337)	15 (ID=336)
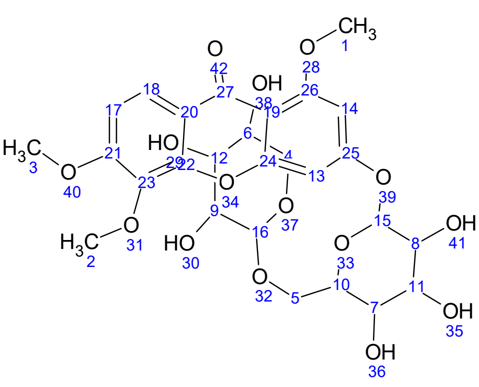	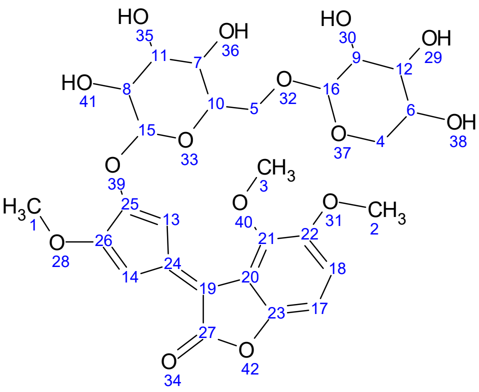	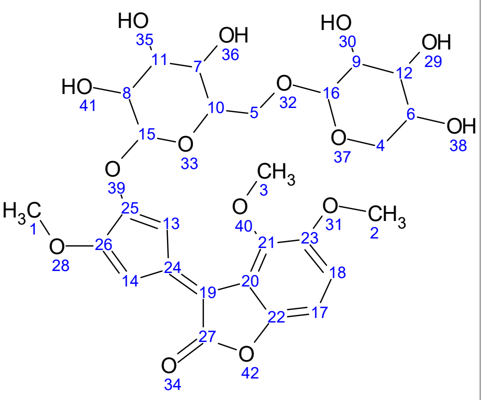
$d_N(^{13}C+^{1}H)$: 6.309	$d_N(^{13}C+^{1}H)$: 6.654	$d_N(^{13}C+^{1}H)$: 6.654
16 (ID=374)	17 (ID=422)	18 (ID=301)
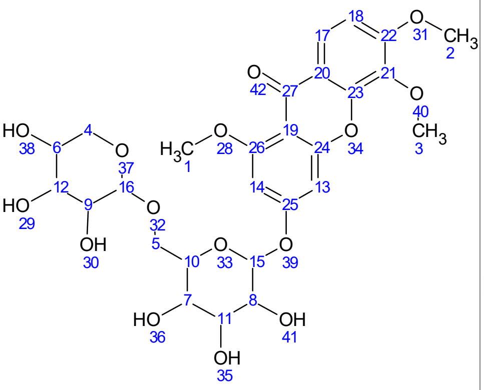	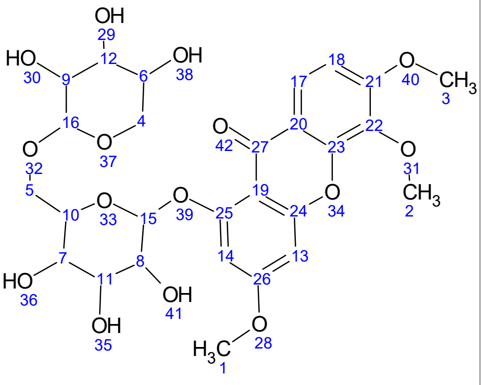	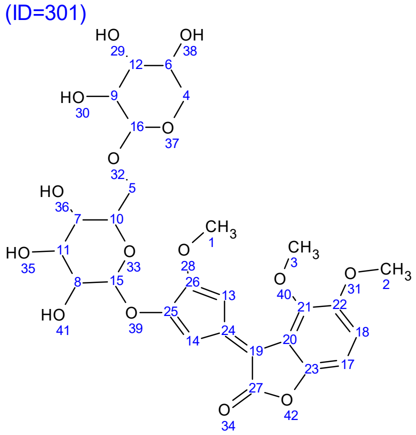
$d_N(^{13}C+^{1}H)$: 6.692	$d_N(^{13}C+^{1}H)$: 6.726	$d_N(^{13}C+^{1}H)$: 6.799

19 (ID=300)

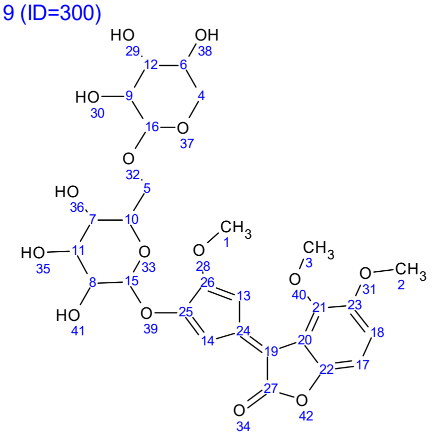

d_N(^{13}C+^{1}H): 6.799

20 (ID=338)

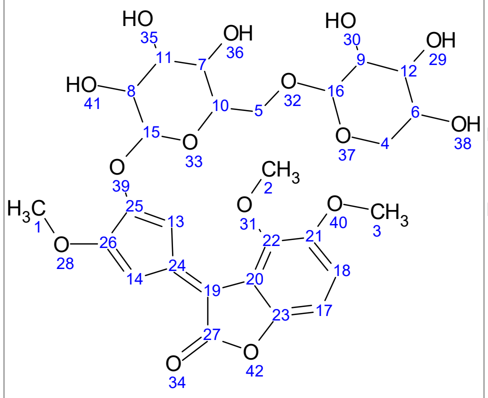

d_N(^{13}C+^{1}H): 7.095

21 (ID=375)

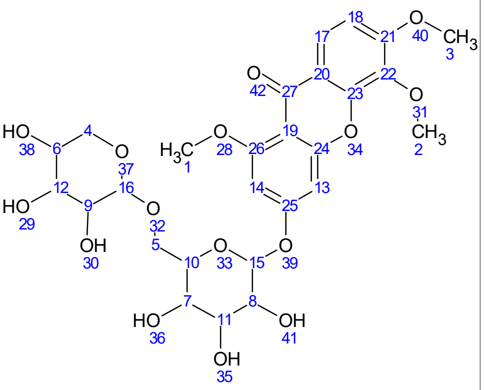

d_N(^{13}C+^{1}H): 7.133

22 (ID=393)

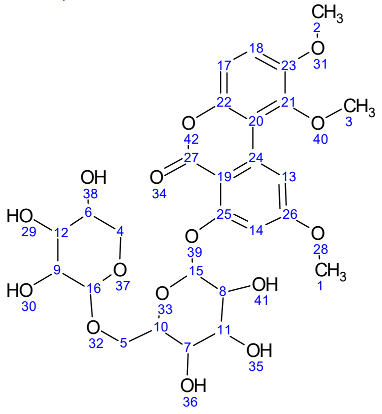

d_N(^{13}C+^{1}H): 7.192

23 (ID=394)

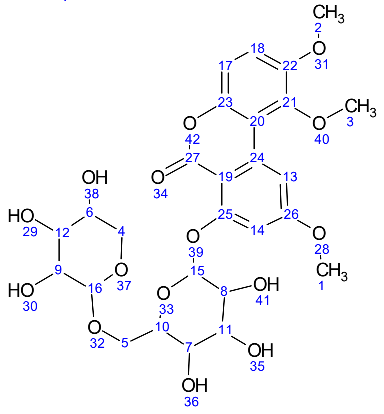

d_N(^{13}C+^{1}H): 7.192

24 (ID=302)

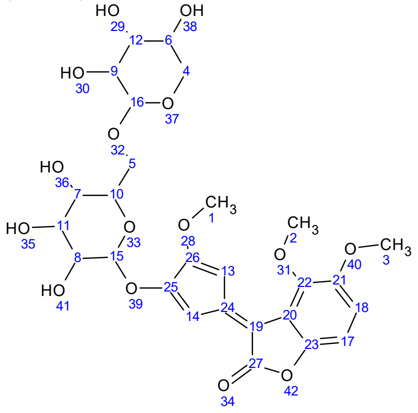

d_N(^{13}C+^{1}H): 7.236

Appendix (Additional Data)

Experimental Spectra Data

standard 1H

Structure Elucidation Report for compound 1 (Page 8)

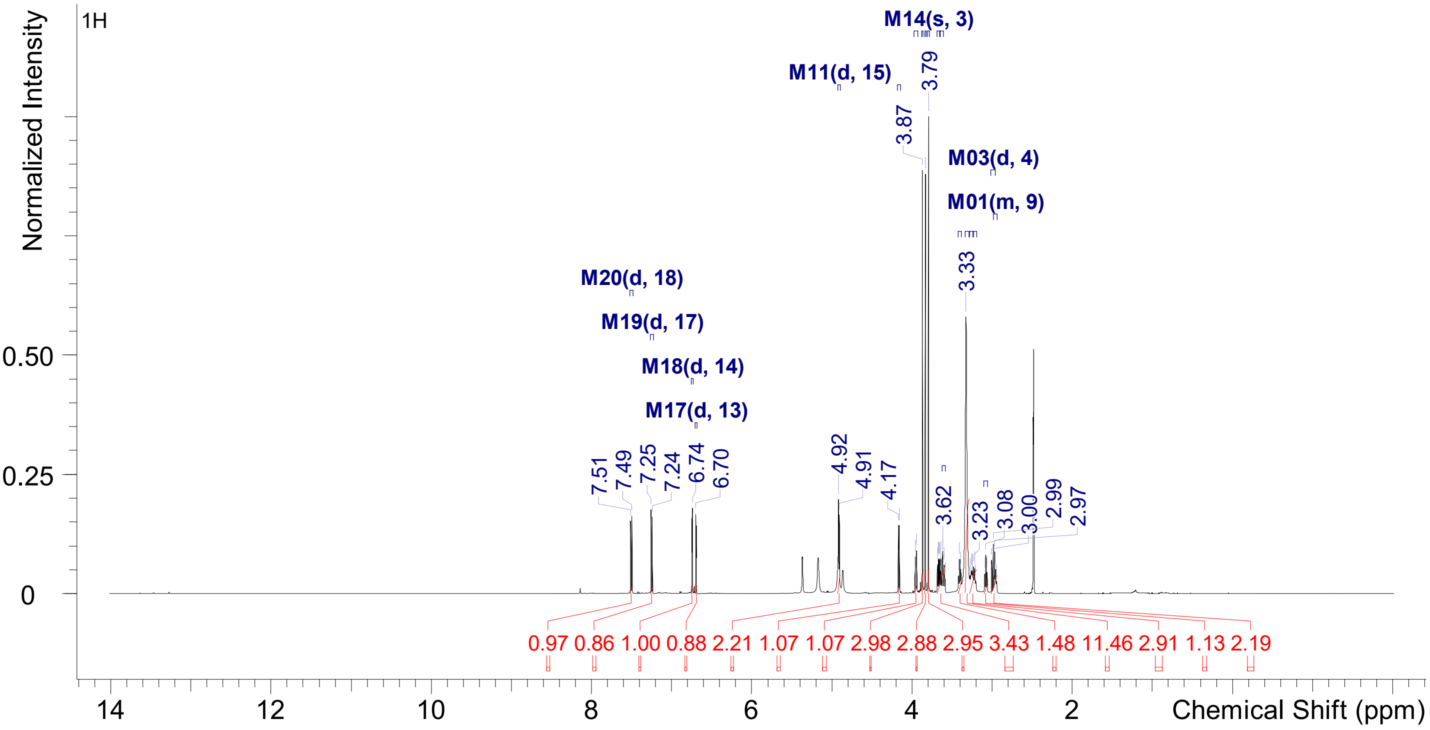

standard 13C

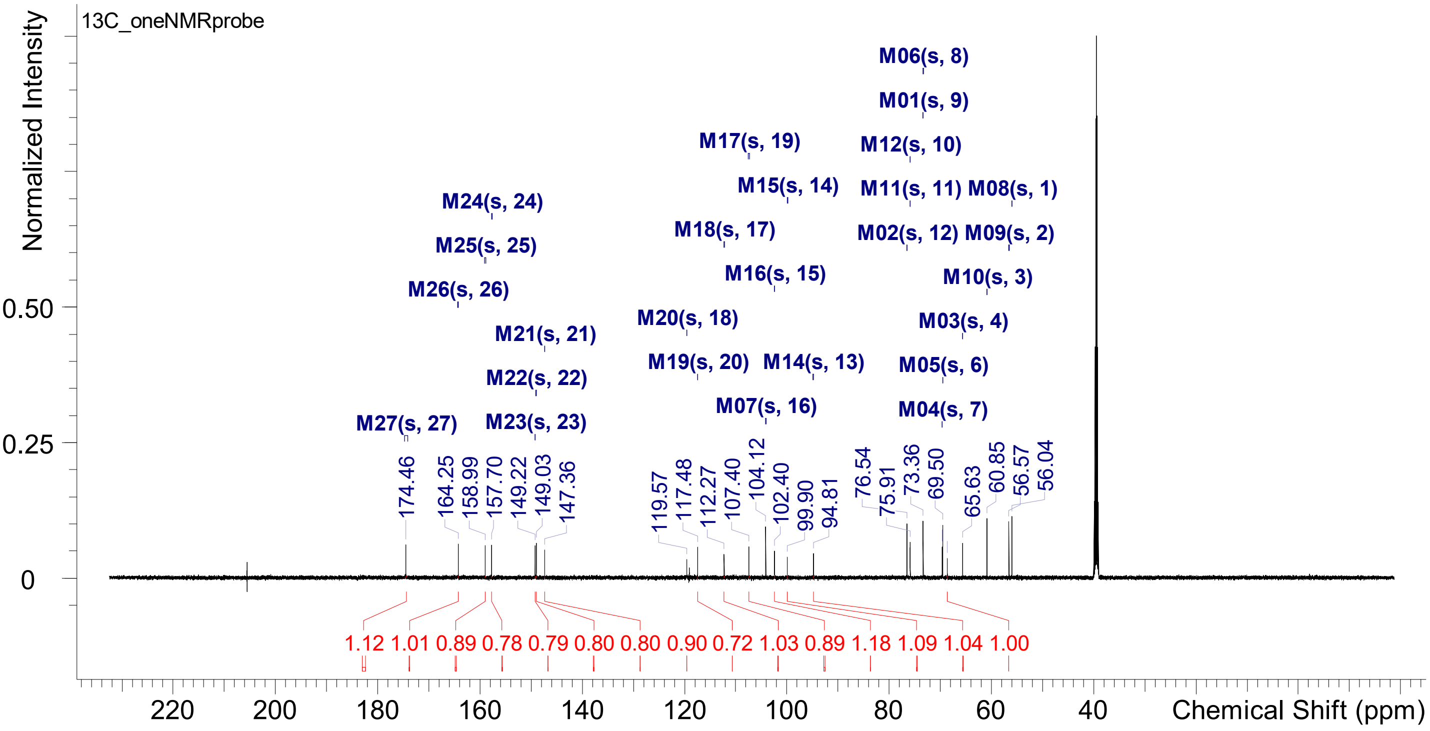

COSY 1H-1H

Structure Elucidation Report for compound 1 Page 9)

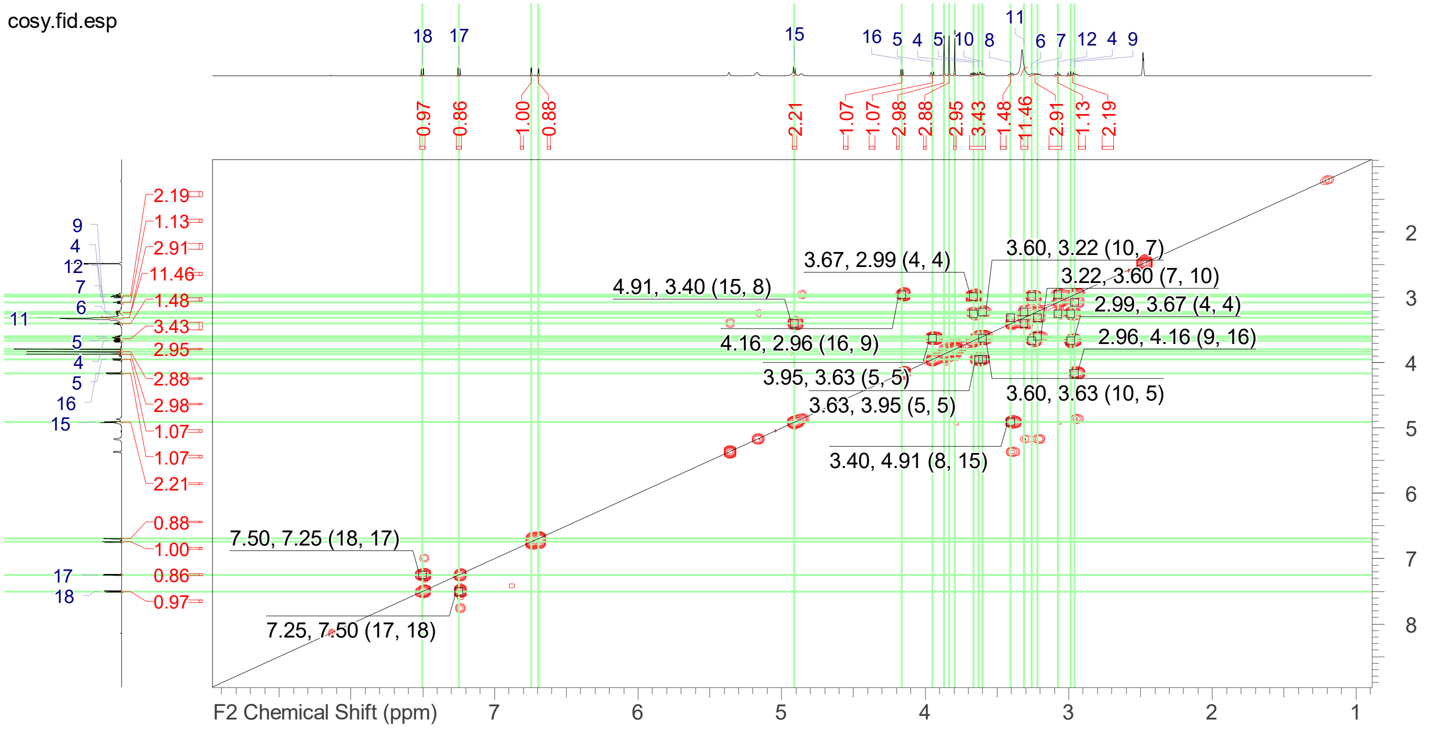

HSQC-DEPT 13C-1H

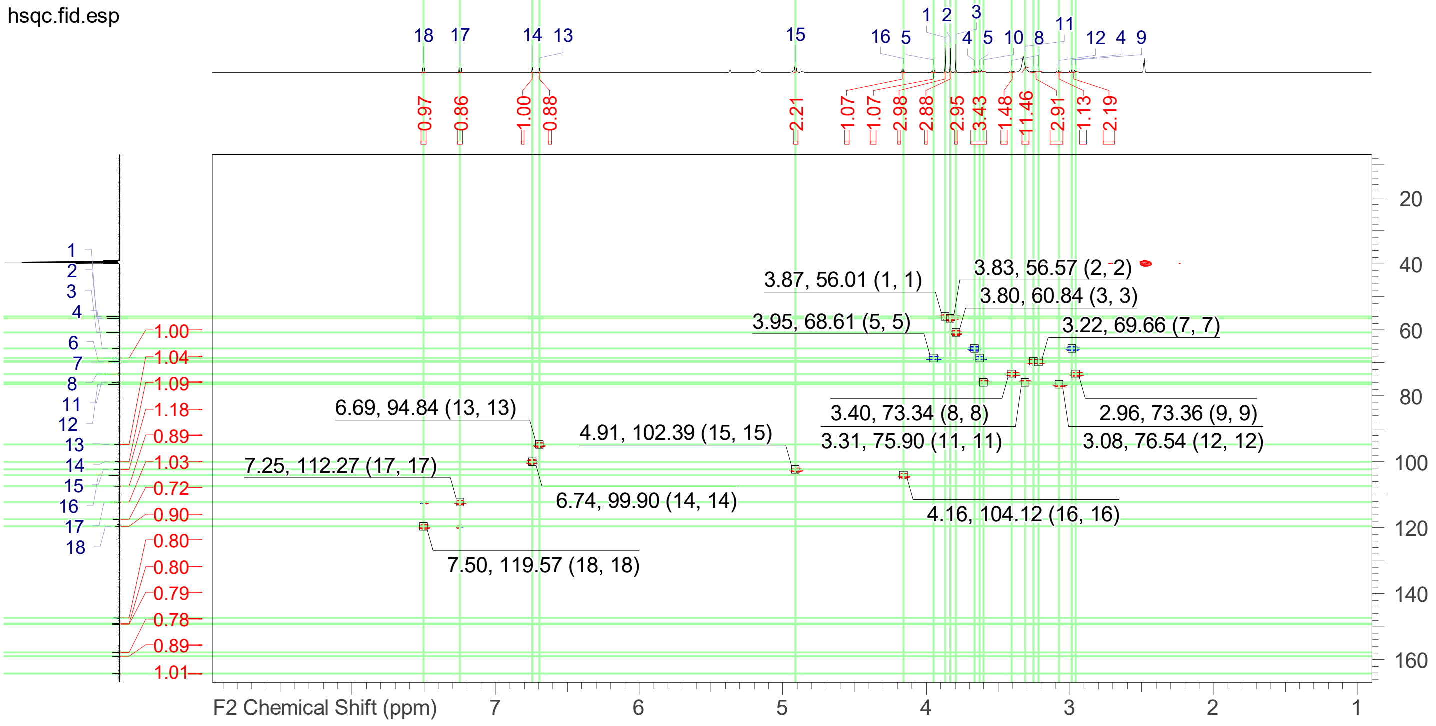

HMBC 13C-1H

Structure Elucidation Report for compound 1 Page 10)

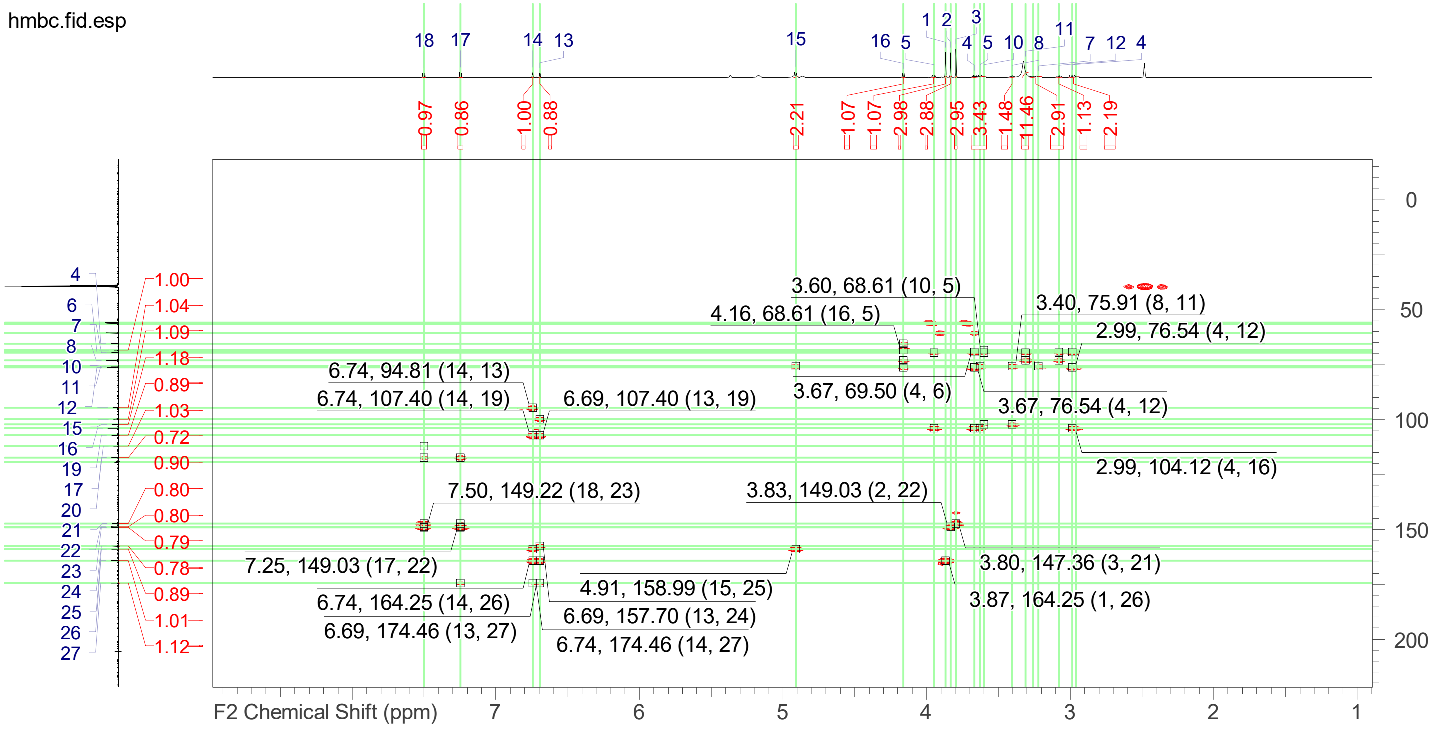

Elucidation Protocol

Start: Structure Generation 07/26/2023 15:46:27

Correlation Spectroscopy Based Structure Generator options:

- Keep Generated Structures = Yes
- Clear Generated Structures List Before Generation = Yes
- Add Structures Already Existing in Generated Structures List = No
- Transfer Spectra to Generated Structures = Yes
- Use Assignment when Removing Duplicates = Yes
- Allow Filter during Generation = Yes
- Use Only 1000 First MCD(s)
- Allow "Fuzzy" Generation = Yes
- "Fuzzy" Generation Options were Determined Automatically
- 2D NMR Spectral Data Must Contain Connectivities = Real spectrum
- Increase Connectivity Length when Merging Connectivities = No
- Allow Bonds between Heteroatoms = No
- Allow Bonds between Heteroatoms of the Same Atom Type = No
- Use NMR Shifts Correlation Table = 2
- Maximum bond multiplicity = 3

MCD #1 (ID = 0): (197109/0) structures have been generated and (484/0) structures stored

197109 structure(s) have been generated by Correlation Spectroscopy Based Generator and 484 structure(s) have been stored.

Generation time: 2 h 09 m 15 s (Check: 5 s, Generation: 2 h 09 m 10 s 340 ms)

No (from No) connectivities have been extended during generation

Finish: Structure Generation 07/26/2023 17:55:43
