# Supplementary material for: Gentiana capitata Buch.–Ham. ex D.Don Cell Suspension Culture as a New Source of Isosaponarin and 3,7,8-Trimethoxy-9-oxo-9H-xanthen-1-yl 6-O-β-D-ribopyranosyl-β-D-allopyranoside and Their Effect on PC-12 Cell Viability
Source: Int J Mol Sci. 2024 Aug 6;25(16):8576. doi: 10.3390/ijms25168576 (PMC11354924; doi:10.3390/ijms25168576)
Supplement: Supplementary file 1 [file ijms-25-08576-s001.zip › Structure Elucidation Report for Compound 2.pdf]

The entire structure elucidation report and the elucidation protocol from the ACD/SE Suite tool (Advanced Chemistry Development, Inc. (ACD/Labs), Toronto, ON, Canada, www.acdlabs.com) for **compound 2**

# Structure Elucidation Report for compound 2

## Initial Data

Composition Restrictions:
Molecular Weight = 0.000-1000.000
Double Bonds Equivalent = 0.00-100.00
Allowed Composition = C(0-100) H(0-100) O(0-20) N(0-10)
Molecular Formula = C27H30O15

Spectral Data:
standard 1H - 12 peaks
merged 1H - 18 peaks
standard 13C - 25 peaks
merged 13C - 25 peaks
COSY 1H-1H - 26 peaks
HSQC-DEPT 13C-1H - 18 peaks
HMBC 13C-1H - 47 peaks

## Most Probable Structure

Following structure has been placed to the first position after spectra calculation

**Structure Elucidation Report for compound 2 Page 2)**

## Carbon Assignment

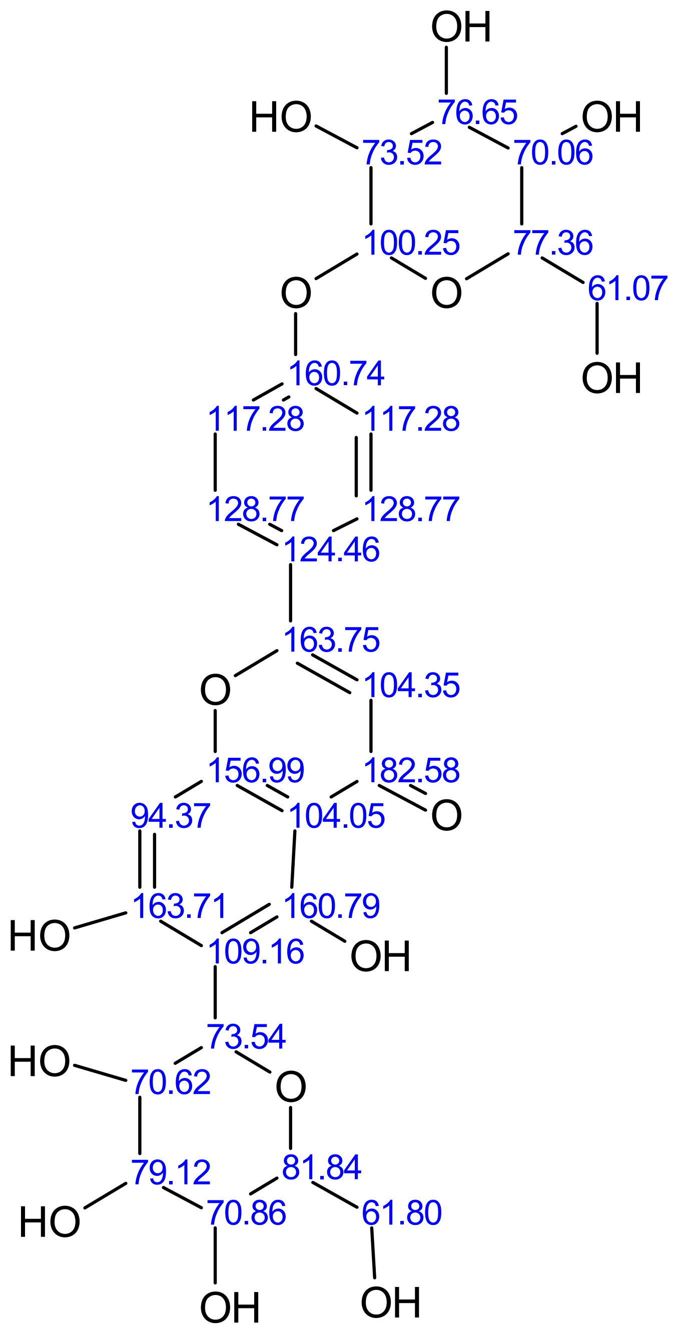


| # | N | Shift (ppm) | Atoms | XHn |
|---|---|---|---|---|
| 1 | 1 | 61.067 | 1 | CH2(t) |
| 2 | 2 | 61.803 | 1 | CH2(t) |
| 3 | 3 | 70.060 | 1 | CH(d) |
| 4 | 4 | 70.621 | 1 | CH(d) |
| 5 | 5 | 70.857 | 1 | CH(d) |
| 6 | 6 | 73.520 | 1 | CH(d) |
| 7 | 7 | 73.539 | 1 | CH(d) |
| 8 | 8 | 76.654 | 1 | CH(d) |
| 9 | 9 | 77.363 | 1 | CH(d) |
| 10 | 10 | 79.122 | 1 | CH(d) |
| 11 | 11 | 81.839 | 1 | CH(d) |
| 12 | 12 | 94.370 | 1 | CH(d) |
| 13 | 13 | 100.246 | 1 | CH(d) |
| 14 | 19 | 104.055 | 1 | C(s) |
| 15 | 14 | 104.350 | 1 | CH(d) |
| 16 | 20 | 109.158 | 1 | C(s) |
| 17 | 15,16 | 117.278 | 2 | CH(d) |
| 18 | 21 | 124.462 | 1 | C(s) |
| 19 | 17,18 | 128.769 | 2 | CH(d) |
| 20 | 22 | 156.989 | 1 | C(s) |
| 21 | 23 | 160.736 | 1 | C(s) |
| 22 | 24 | 160.790 | 1 | C(s) |
| 23 | 25 | 163.710 | 1 | C(s) |
| 24 | 26 | 163.748 | 1 | C(s) |

**Structure Elucidation Report for compound 2 (Page 3)**

## Proton Assignment

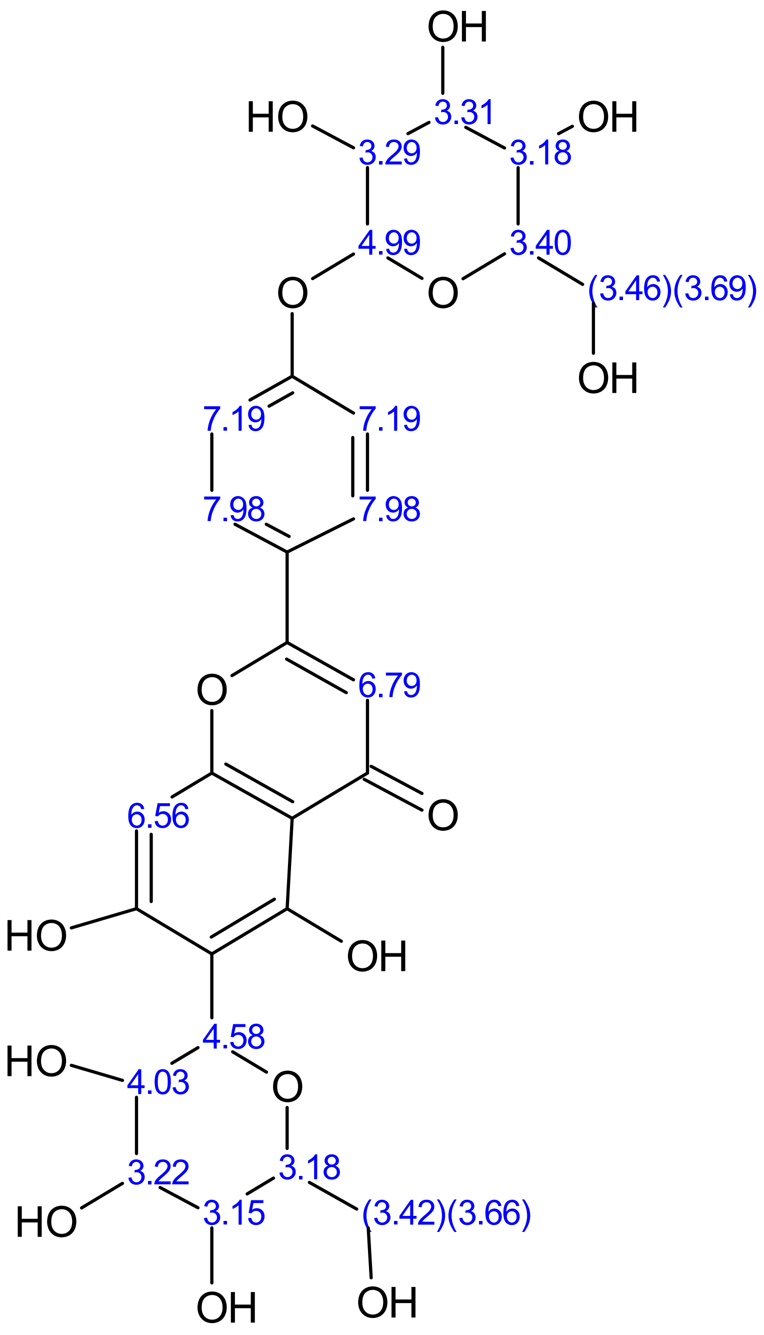


| # | N | Mark | Shift (ppm) | Protons |
|---|---|---|---|---|
| 1 | 5 | | 3.155 | 1 |
| 2 | 11 | | 3.177 | 1 |
| 3 | 3 | | 3.178 | 1 |
| 4 | 10 | | 3.216 | 1 |
| 5 | 6 | | 3.286 | 1 |
| 6 | 8 | | 3.312 | 1 |
| 7 | 9 | | 3.400 | 1 |
| 8 | 2 | | 3.415 | 1 |
| 9 | 1 | | 3.465 | 1 |
| 10 | 2 | | 3.661 | 1 |
| 11 | 1 | | 3.691 | 1 |
| 12 | 4 | | 4.026 | 1 |
| 13 | 7 | | 4.583 | 1 |
| 14 | 13 | | 4.994 | 1 |
| 15 | 12 | | 6.556 | 1 |
| 16 | 14 | | 6.792 | 1 |
| 17 | 15,16 | | 7.189 | 1 |

# Generated Structures

| | | |
|---|---|---|
| 1 (ID=262)<br>The Best Structure<br>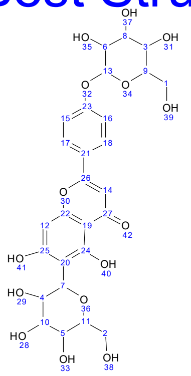 | 2 (ID=331)<br>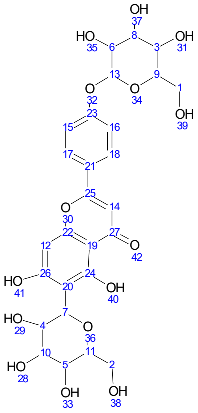 | 3 (ID=107)<br>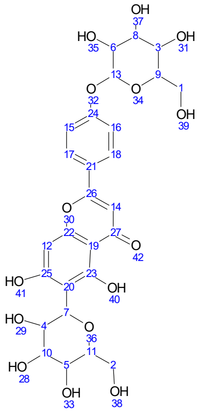 |
| $d_N(^{13}C+^{1}H)$: 3.779 | $d_N(^{13}C+^{1}H)$: 3.782 | $d_N(^{13}C+^{1}H)$: 3.783 |
| 4 (ID=166)<br>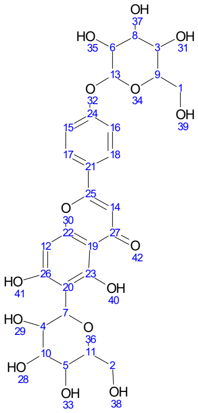 | 5 (ID=327)<br>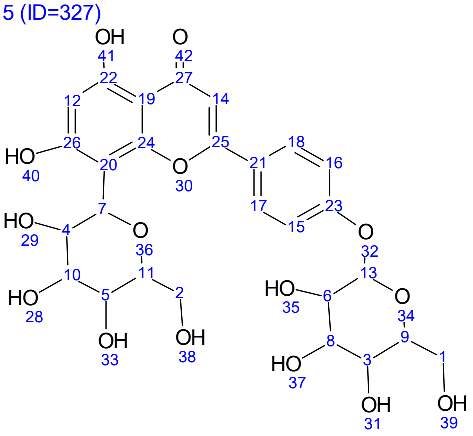 | 6 (ID=162)<br>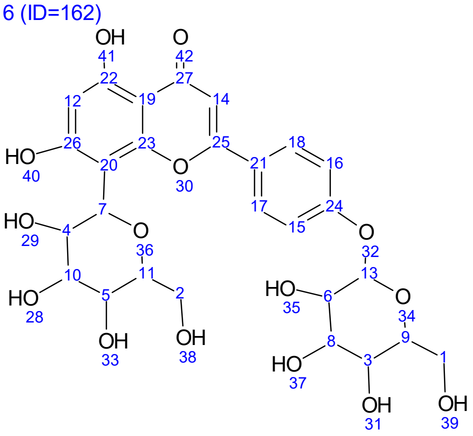 |
| $d_N(^{13}C+^{1}H)$: 3.786 | $d_N(^{13}C+^{1}H)$: 4.339 | $d_N(^{13}C+^{1}H)$: 4.339 |

## Structure Elucidation Report for compound 2 Page 5)

7 (ID=258)

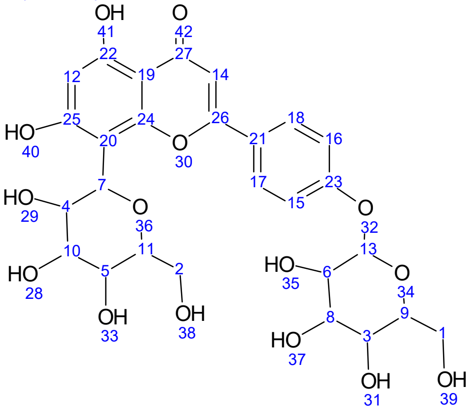

$d_N(^{13}C+^{1}H)$: 4.341

8 (ID=103)

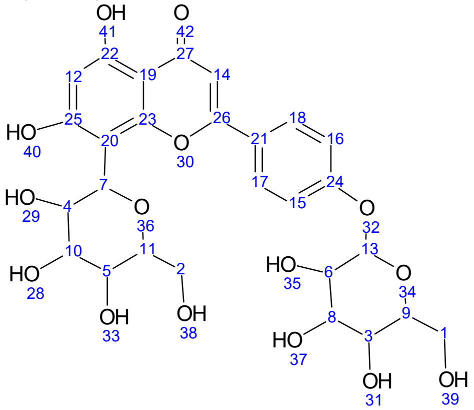

$d_N(^{13}C+^{1}H)$: 4.341

9 (ID=257)

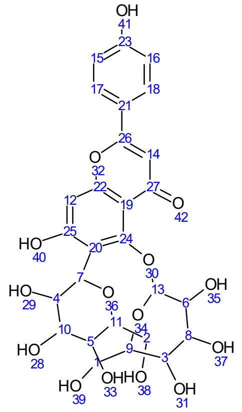

$d_N(^{13}C+^{1}H)$: 4.889

10 (ID=102)

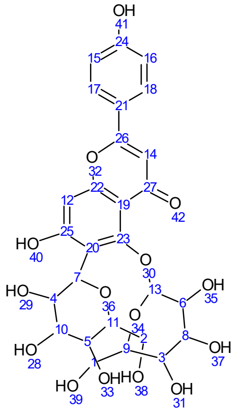

$d_N(^{13}C+^{1}H)$: 4.889

11 (ID=326)

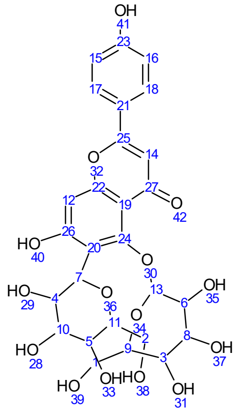

$d_N(^{13}C+^{1}H)$: 4.892

12 (ID=161)

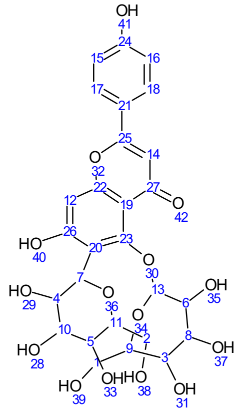

$d_N(^{13}C+^{1}H)$: 4.892

# Structure Elucidation Report for compound 2 Page 6)

13 (ID=255)

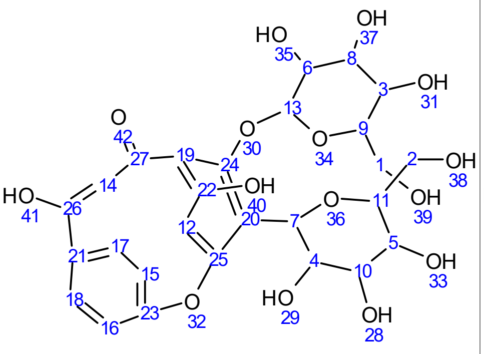

$d_N(^{13}C+^{1}H)$: 6.518

14 (ID=100)

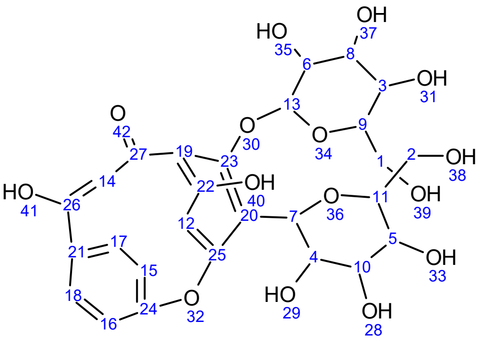

$d_N(^{13}C+^{1}H)$: 6.518

15 (ID=324)

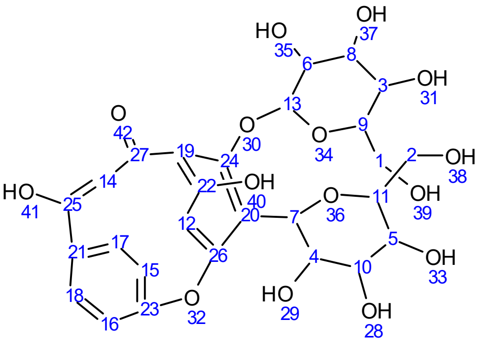

$d_N(^{13}C+^{1}H)$: 6.521

16 (ID=159)

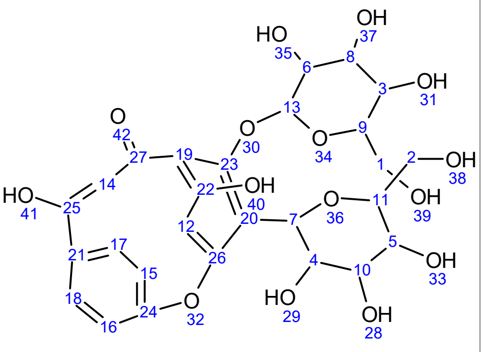

$d_N(^{13}C+^{1}H)$: 6.521

17 (ID=330)

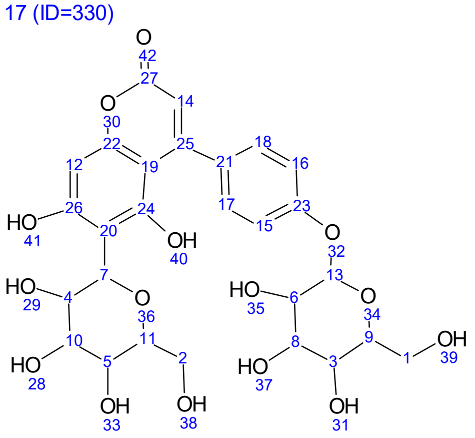

$d_N(^{13}C+^{1}H)$: 6.806

18 (ID=165)

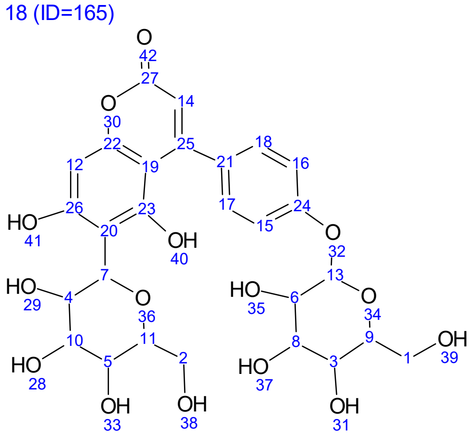

$d_N(^{13}C+^{1}H)$: 6.806

19 (ID=261)

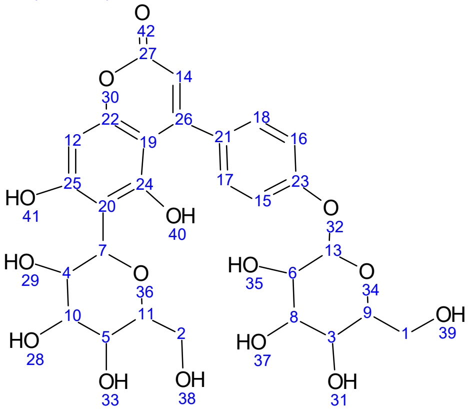

$d_N(^{13}C+^{1}H)$: 6.806

20 (ID=106)

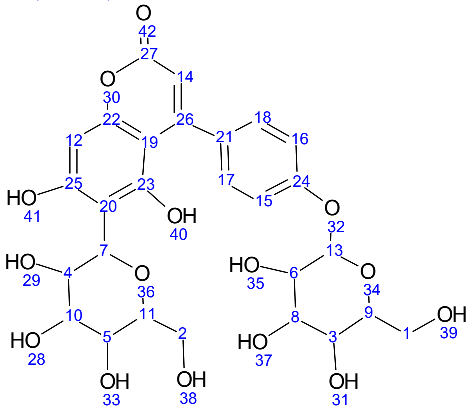

$d_N(^{13}C+^{1}H)$: 6.806

21 (ID=337)

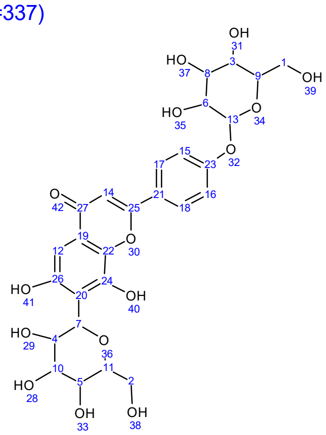

$d_N(^{13}C+^{1}H)$: 7.212

22 (ID=270)

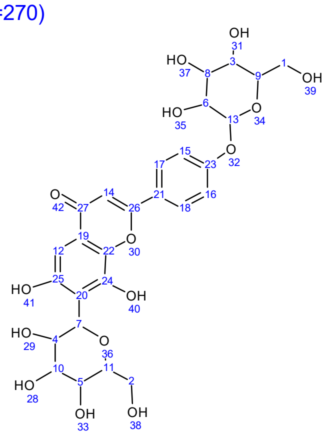

$d_N(^{13}C+^{1}H)$: 7.212

23 (ID=172)

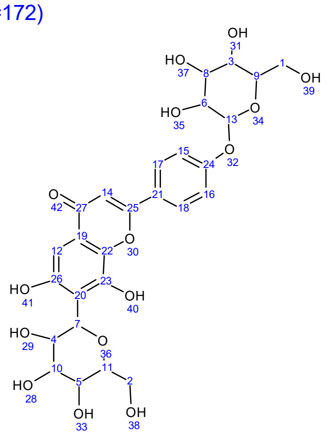

$d_N(^{13}C+^{1}H)$: 7.212

24 (ID=113)

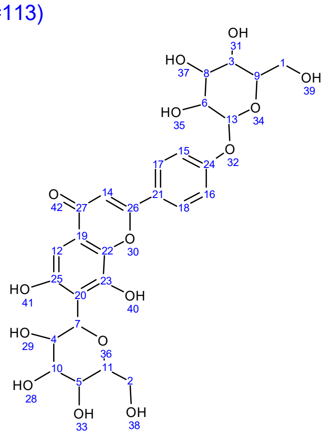

$d_N(^{13}C+^{1}H)$: 7.212

# Appendix (Additional Data)

## Experimental Spectra Data

### standard 1H

**Structure Elucidation Report for compound 2 Page 8)**

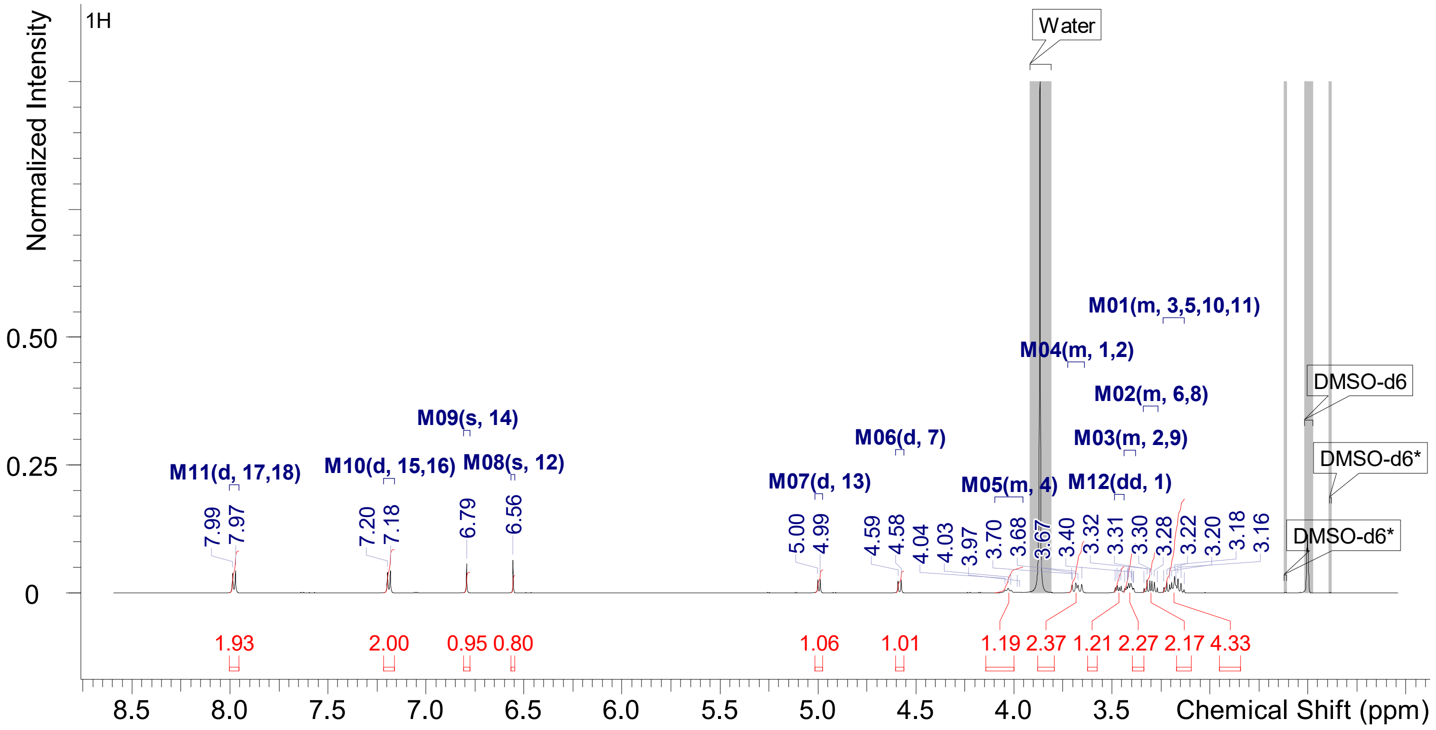


## standard 13C

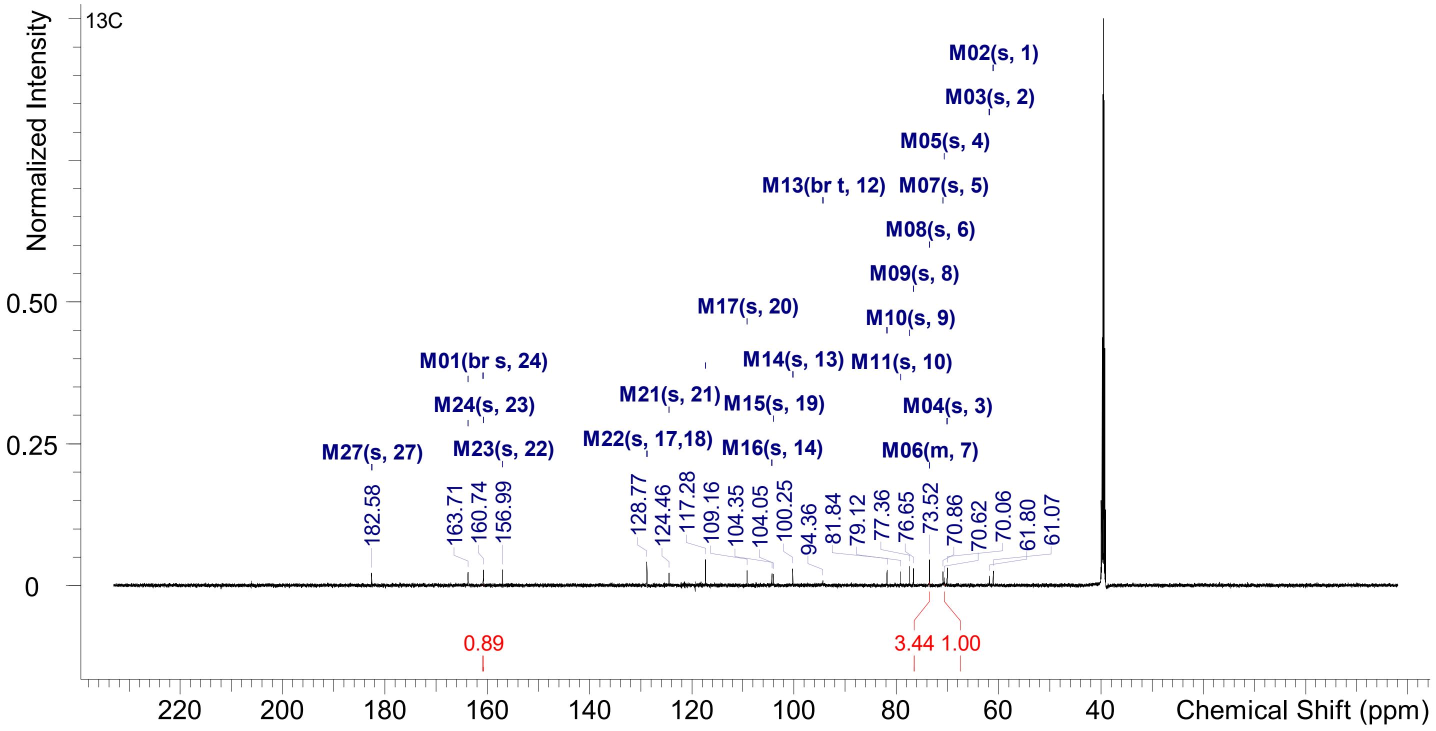


## COSY 1H-1H

# Structure Elucidation Report for compound 2 Page 9)

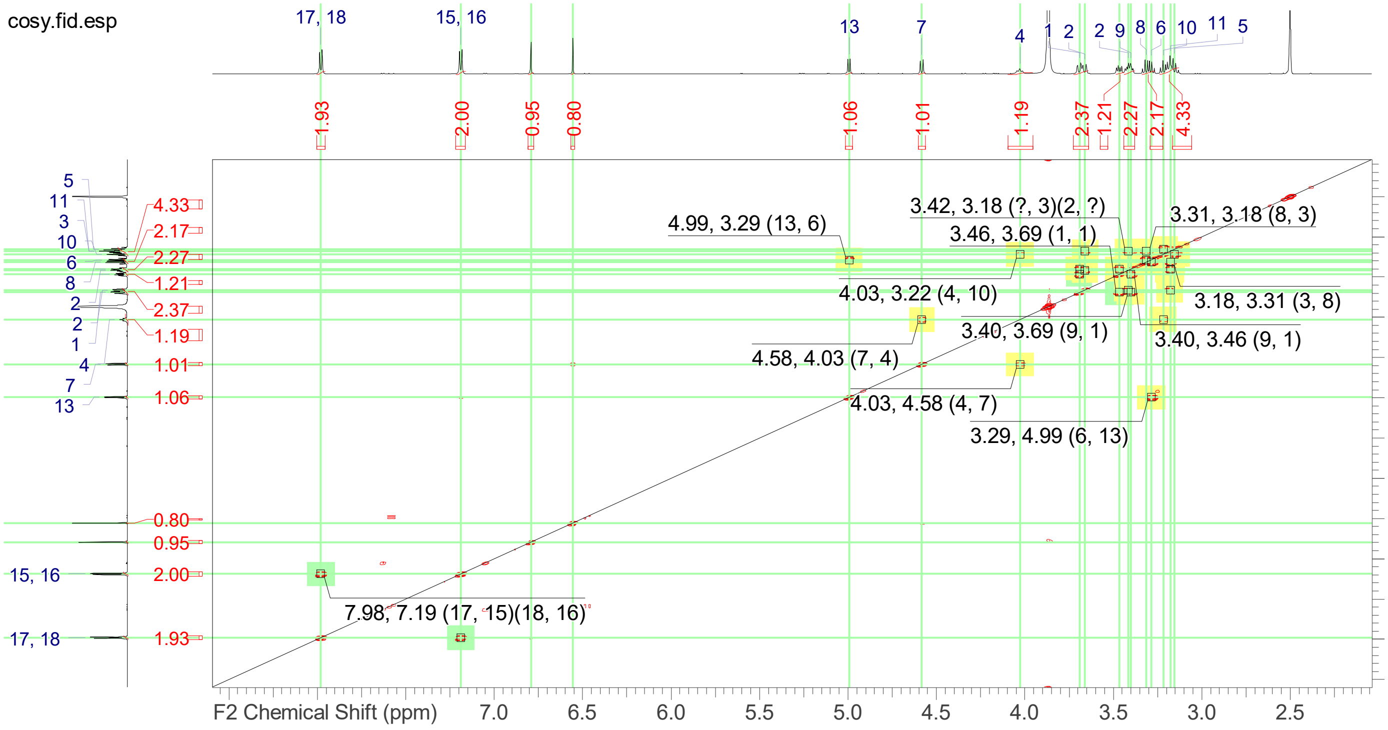


## HSQC-DEPT 13C-1H

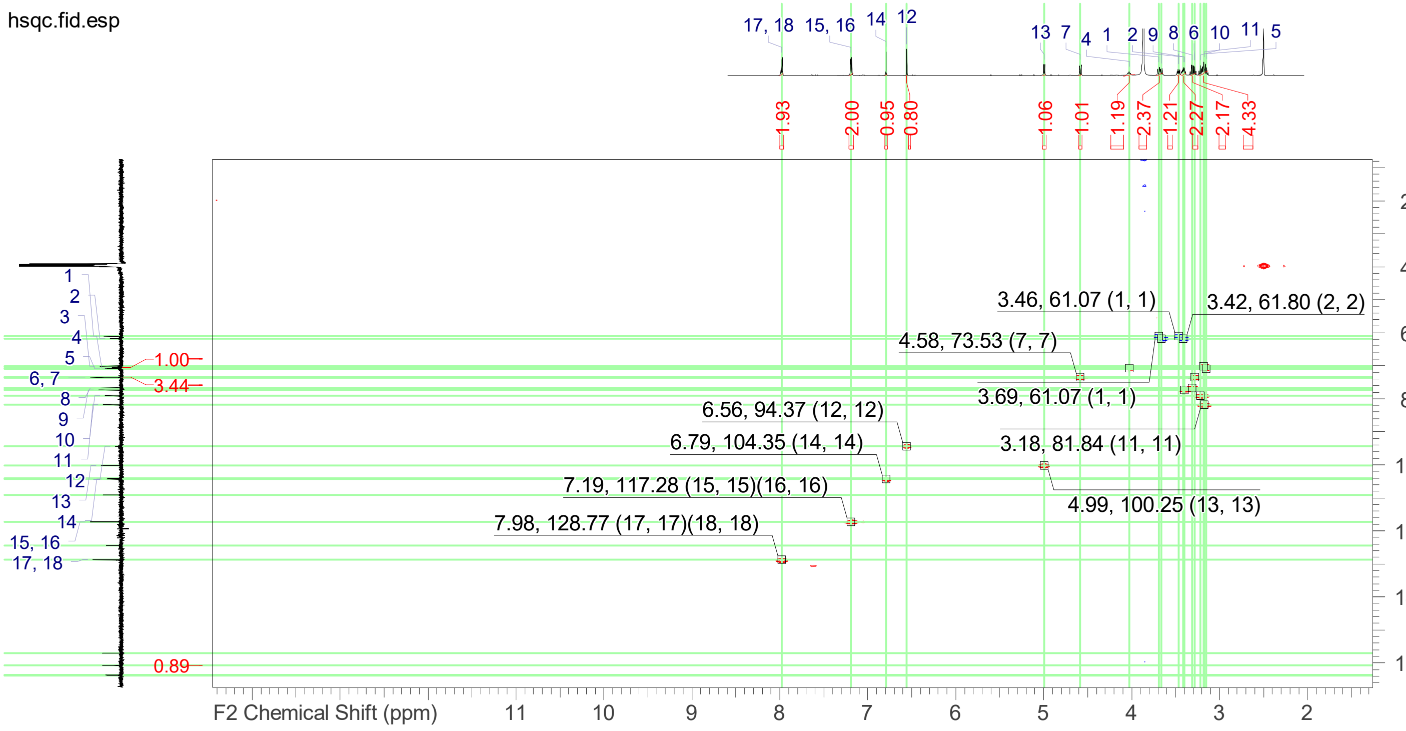


## HMBC 13C-1H

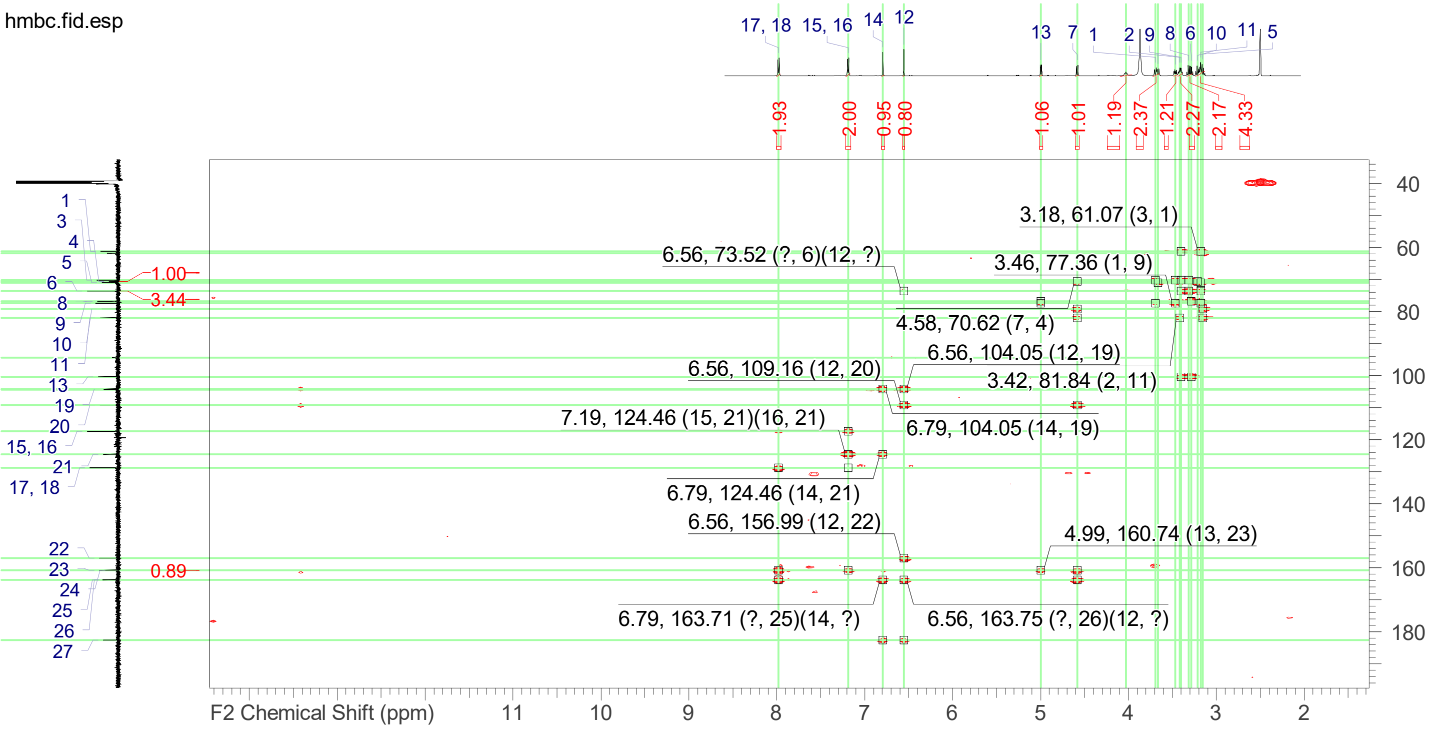


# Elucidation Protocol

Start: Structure Generation 09/22/2023 10:24:27
Correlation Spectroscopy Based Structure Generator options:

- Keep Generated Structures = Yes
- Clear Generated Structures List Before Generation = Yes
- Add Structures Already Existing in Generated Structures List = No
- Transfer Spectra to Generated Structures = Yes
- Use Assignment when Removing Duplicates = Yes
- Allow Filter during Generation = Yes
- Use Only 1000 First MCD(s)
- Allow "Fuzzy" Generation = Yes
- "Fuzzy" Generation Options were Determined Automatically
- 2D NMR Spectral Data Must Contain Connectivities = Real spectrum
- Increase Connectivity Length when Merging Connectivities = No
- Allow Bonds between Heteroatoms = No
- Allow Bonds between Heteroatoms of the Same Atom Type = No
- Use NMR Shifts Correlation Table = 2
- Maximum bond multiplicity = 3

MCD #1 (ID = 0): (779319/0) structures have been generated and (430/0) structures stored
779319 structure(s) have been generated by Correlation Spectroscopy Based Generator and 430 structure(s) have been stored.
Generation time: 121 h 47 m 01 s (Check: 0 s, Generation: 121 h 47 m 00 s 969 ms)
No (from No) connectivities have been extended during generation
Generation has been interrupted by the user (stop button pressed)
Finish: Structure Generation 09/27/2023 12:11:30
